# Understanding stigma consciousness: A multilevel analysis across diverse stigmatized groups

**DOI:** 10.1111/bjso.70072

**Published:** 2026-03-23

**Authors:** Joelle‐Cathrin Flöther, Juliane Degner

**Affiliations:** ^1^ Department of Psychology University Hamburg Hamburg Germany

**Keywords:** discrimination experiences, group status, stigma consciousness, stigmatization, stigmatized groups

## Abstract

Members of stigmatized groups differ in how they perceive, reflect on and cope with their stigmatized group status. One potential variable explaining these differences is stigma consciousness: the extent to which individuals expect to be stigmatized based on their group membership (Pinel, *J. Pers. Soc. Psychol*., 76, 1999, 114). However, theorizing and research on predictors of interindividual and between‐group variance in stigma consciousness are limited so far. The present research systematically investigated several variables potentially contributing to differences in stigma consciousness at the individual, stigma, and group levels. Multilevel regression modelling using data collected from *N* = 3969 members of 18 different stigmatized groups revealed that stigma consciousness varies primarily between individuals and less between groups and is largely predicted by individual‐level factors, including discrimination experiences, ingroup identification and ideological beliefs. Our findings help to refine the conceptualization of stigma consciousness as an individual psychological characteristic, shaped primarily by individual perceptions and experiences rather than group memberships. Future research directions are discussed, highlighting further individual‐level predictors as well as outcomes of stigma consciousness.

## INTRODUCTION

A large body of social psychological research has documented that stigmatization has adverse cognitive, behavioural and interpersonal consequences, creating significant challenges for individuals belonging to stigmatized groups in daily life and intergroup relations (Costa et al., 2022; Schmitt et al., 2014). However, individuals vary in how they perceive, anticipate and respond to stigmatization. One potential concept that might explain these differences is stigma consciousness: The extent to which individuals expect to be stigmatized based on their social group (Pinel, 1999). Research has documented that differences in stigma consciousness, both within and across groups, can moderate the impact of stigmatization (Bazemore et al., 2010; Pinel, 2002, 2004). Despite this potential impact, little theoretical and empirical work has examined the variability of stigma consciousness. The present research aims to systematically investigate factors that account for individual differences in stigma consciousness. Drawing on Pinel's conceptualization (1999), and broader theorizing and empirical work on social identity (Tajfel & Turner, 1979) and related processes (Jost & Banaji, 1994) as well as psychological and sociological work on stigmatization (Link & Phelan, 2001; Pachankis et al., 2018; Quinn & Earnshaw, 2011), we identified several potential variables that may operate at the individual, stigma and group level. We investigate these factors relying on the perspectives of members of 18 stigmatized groups in the United States employing a multilevel approach to refine our understanding of stigma consciousness and its predictors on various levels.

We begin with an overview of the theoretical conceptualization and empirical findings on stigma consciousness before turning to potential predictors of individual and group differences in stigma consciousness.

### Conceptualizing stigma consciousness

Pinel (1999) proposed that stigmatized group members differ in how they perceive and experience their stigmatized group status and that these differences shape the way in which stigmatization affects them. This variability is referred to as *stigma consciousness*: the extent to which individuals expect to be stereotyped based on their ingroup membership and believe that their stigmatized group status shapes interactions with outgroup members (Pinel, 1999). Stigma consciousness is distinct from related constructs within social psychological literature, such as stereotype consciousness—the awareness that stereotypes exist in the prevailing society (McKown & Weinstein, 2003)—and discrimination experiences—the frequency of incidents perceived as discrimination (Williams et al., 1997). High and low stigma conscious individuals do not differ in their awareness or recognition of stigmatization, but rather in the degree to which they perceive this stigmatization to affect them personally and the extent to which they focus on their ingroup status (Pinel, 1999, 2004): while high stigma conscious individuals are more likely to experience social interactions through the lens of group membership and anticipate its negative consequences, those low in stigma consciousness are less inclined to attribute their experiences to their group and expect less impact of stigmatization on their daily lives (Bazemore et al., 2010; Pinel, 2002, 2004; Pinel & Paulin, 2005).

Moreover, stigma consciousness impacts how individuals perceive and process their ingroup status and to what extent a stigmatized status affects them on various levels (Pinel, 1999, 2004). Empirical findings across a diverse variety of stigmatized groups including women, ethnic and sexual minority groups, support this view: High stigma conscious individuals feel more self‐conscious, less self‐efficient and more disrespected by others (Daley & Rappolt‐Schlichtmann, 2018; Lee & Brown, 2023; Moore & Tangney, 2017; Pinel, 1999; Pinel & Paulin, 2005), are more inclined to attribute negative evaluations to discrimination (Pinel, 2004; Wang et al., 2012), and report more frequent discrimination experiences (Pinel, 1999). Additionally, high stigma conscious individuals report more negative emotions and lower well‐being and self‐esteem (Bazemore et al., 2010; Berghe et al., 2010; Lee & Brown, 2023; Lewis et al., 2003, 2006; Magallares et al., 2016; Pinel, 2004; Pinel et al., 2005; Pinel & Paulin, 2005; Reilly et al., 2019; Wang et al., 2012). Concerning behavioural consequences, research suggests that stigma consciousness is positively related to academic disengagement and performance declines (Brown & Lee, 2005; Brown & Pinel, 2003; Daley & Rappolt‐Schlichtmann, 2018; Pinel et al., 2005). Regarding interpersonal relationships, high stigma conscious individuals tend to express more distrust in people (Pinel, 1999), to act critically towards outgroup members if they believe others hold stereotypes about the ingroup (Pinel, 2002) and to avoid interactions with outgroup members (Moore & Tangney, 2017; Pinel, 1999; Son & Shelton, 2011). Additionally, stigma consciousness impacts coping behaviour and how individuals navigate consequences of stigmatization, with both positive and negative effects: On the one hand, high stigma consciousness is related to identity concealment and avoidance of situations with stigmatization potential, limiting opportunities to challenge and change group stereotypes (Nouvilas‐Pallejà et al., 2018; Pinel, 1999). On the other hand, high stigma conscious individuals tend to attribute negative evaluations more likely to discrimination thus safeguarding individual self‐esteem (Pinel, 1999, 2004) and report more adaptive coping strategies (Nouvilas‐Pallejà et al., 2018; Wang et al., 2012).

This substantial evidence marks stigma consciousness as a key variable that impacts cognitive, behavioural and interpersonal outcomes, moderating the effects of stigmatization. To explain why individuals perceive and respond to stigmatization differently, it thus appears crucial to examine stigma consciousness and the factors contributing to its variation. To date, however, potential correlates of the variability in stigma consciousness remain largely unexplored, both theoretically and empirically. We aim to address this gap by systematically investigating predictors of stigma consciousness across a variety of stigmatized groups, thereby complementing theorizing and research.

### Predictors of stigma consciousness—a multilevel approach

While no theoretical account directly addresses predictors of stigma consciousness, we draw on related theoretical perspectives and empirical findings within the stigmatization and social identity literature, which suggest that differences in stigma consciousness may arise from both individual dispositions, stigma characteristics and further group‐level factors (Pinel, 1999, 2004; Pinel et al., 2005). Albeit Pinel originally conceptualized stigma consciousness strictly as an individual variable, we nevertheless consider it informative to additionally examine stigma consciousness at the group level. Prior work has documented that stigmatized groups vary systematically in average stigma consciousness and its psychological outcomes (Brown & Lee, 2005; Burgess et al., 2018), yet it remains unclear whether such differences arise from individual‐ or group‐level factors. On the one hand, observed between‐group differences may reflect variation in member composition, that is, differences in the characteristics of ingroup members, without any influence of the group context. On the other hand, they may reflect contextual variation in how stigmatized groups are perceived and treated within society, with shared group characteristics shaping stigma consciousness beyond individual factors. Indeed, prior research documents between‐group differences in the type and frequency of discrimination experiences (Charlesworth et al., 2023), socioeconomic status hierarchies (Kollar & Scherer, 2025), group evaluation (Essien et al., 2021) and stigma characteristics (Pachankis et al., 2018). Contextual factors are demonstrated to influence stigmatization perceptions and experiences (Essien et al., 2021; Pachankis et al., 2018), which may, in turn, impact stigma consciousness. Accordingly, while individual differences are central, stigma consciousness may also be shaped by group‐level differences, underscoring the importance of accounting for between‐group variability. We therefore adopt a multilevel framework—spanning individual, stigma and group characteristics—to provide a more comprehensive account of stigma consciousness.

#### Individual‐level characteristics

The most frequently addressed individual‐level predictor of stigma consciousness refers to *discrimination experiences*. Pinel (1999) argued that frequent past discrimination fosters expectations of future discrimination, manifesting as stigma consciousness. There is indeed empirical support for a robust positive association between stigma consciousness and discrimination experiences across diverse stigmatized groups (Bird & Bogart, 2001; Brewster et al., 2016; Degner et al., 2021; Lee & Brown, 2023; Magallares et al., 2016; Molero et al., 2013; Nouvilas‐Pallejà et al., 2018; Pinel, 1999; Quinn et al., 2020). Notably, the nature of this relation remains ambiguous, as most studies are cross‐sectional, thereby limiting conclusions about directionality. Pinel (1999, 2004) proposed two pathways: (a) past experiences of discrimination heighten stigma consciousness, and (b) high stigma consciousness increases recognition of discrimination, as individuals attuned to structural bias are more likely to interpret ambiguous experiences as discriminatory. We treat discrimination experiences as a potential predictor of stigma consciousness, expecting to replicate prior positive correlations while acknowledging the link may be bidirectional.

Our second individual‐level predictor draws on Social Identity Theory (SIT; Tajfel & Turner, 1979), which posits that social identities fundamentally shape individuals' self‐concepts, social perceptions, attitudes and behaviours. As such, the degree of *social identification*—how strongly a person aligns with and feels connected to their social ingroup (Leach et al., 2008; Postmes et al., 2012)—may be related to stigma consciousness because highly identified individuals are more likely to reflect on their ingroup and become increasingly aware of its stigmatization. Pinel's (1999) theorizing supports these assumptions: While she did not consider (high) social identification to be necessary for developing (high) stigma consciousness, she assumed that individuals with a stronger sense of belonging to their group may also exhibit higher stigma consciousness.

To date, empirical research on the link between stigma consciousness and social identification is scarce and inconclusive: Stigma consciousness seems positively linked to ingroup identification among racial and religious minorities (Pasek, 2015; Wilton et al., 2012). For sexual minorities, however, higher stigma consciousness related to greater identity centrality but also increased identity concealment, suggesting simultaneous engagement with and distancing from the ingroup (Nouvilas‐Pallejà et al., 2018). Since the variability in prior findings may, in part, be explained by between‐group differences in the relationship between social identification and stigma consciousness, we expect a positive association between social identification and stigma consciousness and explore whether there are divergent correlation patterns across groups.

SIT also indirectly suggests another individual‐level predictor of stigma consciousness: *ideological beliefs*, specifically, the extent to which individuals perceive status differences between groups as (il)legitimate, influence their responses to low ingroup status (Tajfel & Turner, 1979). A similar notion is put forth in System Justification Theory (SJT; Jost & Banaji, 1994), which argues that low status groups' endorsement or rejection of negative ingroup stereotypes depends on whether individuals view social structures as fair or unjust, which is often linked to ideological orientation along a traditional/conservative‐progressive/liberal dimension (De Oliveira Santos & Jost, 2024). Accordingly, stigmatized group members who view society as fair tend to accept negative ingroup stereotypes and disadvantage as justified, whereas those who view society as illegitimate are more likely to recognize ingroup disadvantage as stigmatization, which, in turn, can heighten stigma consciousness. Indeed, prior research has documented that stigmatized group members who perceive the prevailing system as unfair or illegitimate, or who hold more liberal orientations, report more discrimination experiences (Major et al., 2002; Osborne & Sibley, 2013; Owuamalam et al., 2016) and are more likely to attribute low ingroup status to stigmatization and systemic injustice (Degner et al., 2021). We therefore include *system justification beliefs* (i.e., the degree to which individuals perceive the societal system as fair and legitimate) and *ideological orientation* (i.e., the individual's position on a spectrum ranging from liberal to conservative) as predictors of stigma consciousness.

The final individual‐level variable we consider relevant for stigma consciousness draws on theoretical work within social identity and stigmatization frameworks: The Model of Concealable Stigmatized Identities (CSI; Quinn & Earnshaw, 2011) proposes that *internalized stigma*, that is, the endorsement of negative ingroup stereotypes as true to the self, is linked to the anticipation of stigmatization. Stigma consciousness and stigma internalization are conceptualized as two distinctive but related processes (Corrigan & Watson, 2002; Earnshaw & Quinn, 2012; Pinel, 1999; Quinn & Earnshaw, 2011): Individuals who internalize ingroup stigma are likely to anticipate others to apply this stigma to themselves as well. Supporting this assumption, past research has mainly documented positive correlations between internalized stigma and stigma consciousness (Berghe et al., 2010; Bunn et al., 2007; Lewis et al., 2003, 2006; Quinn & Earnshaw, 2011; Turan et al., 2017). However, such expectations may also arise without believing ingroup stereotypes to be true for the ingroup or self; rather, individuals may expect stigmatization based on prior experiences, even while explicitly rejecting negative ingroup beliefs. We therefore explore whether and to what extent differences in internalized stigma are associated with differences in stigma consciousness.

#### Stigma‐level characteristics

Our second level of theoretical consideration about correlates of stigma consciousness examines characteristics of the specific stigma attached to different social categories. Prior work has classified stigma along various dimensions that shape individuals' perceptions of their stigmatized status (Jones et al., 1984; Pachankis et al., 2018). We propose that, by impacting the frequency and nature of discrimination experiences, these stigma characteristics may further affect stigma consciousness.

We rely on the taxonomy proposed by Pachankis et al. (2018) distinguishing six stigma dimensions: *concealability* (i.e., the extent to which a stigma is visible to others), *origin* (i.e., the extent to which a stigma is perceived as accidentally or deliberately caused), *course* (i.e., the extent to which a stigma is perceived as stable over time), *disruptiveness* (i.e., the extent to which a stigma is perceived as interfering with social interactions), *aesthetics* (i.e., the extent to which a stigma is perceived as unappealing) and *peril* (i.e., the extent to which a stigma is perceived as dangerous).

Most research addressing the association of stigma characteristics and stigmatization has focused on concealability. For example, the CSI Model (Quinn & Earnshaw, 2011) posits that individuals with concealable identities are aware of ingroup stereotypes and anticipate stigmatization if their identity is revealed, leading them to conceal it to avoid negative treatment. Research with sexual minorities and individuals with mental illnesses, indeed, indicates a link between stigma consciousness and greater identity concealment (Abbott & Mollen, 2018; Lewis et al., 2003; Nouvilas‐Pallejà et al., 2018; Quinn & Chaudoir, 2009; Reinka et al., 2020). Notably, a reversed correlation between stigma consciousness and concealability is also plausible: Individuals who regard their stigmatized identity as successfully concealed may exhibit lower stigma consciousness, as they do not expect stigmatization to affect social interactions.

To our knowledge, no research has examined potential links between other stigma characteristics and stigma consciousness, with the notable exception of the study by Pachankis et al. (2018), comparing multiple stigmatized groups. Their results indicate that the more uncontrollable, unstable, disruptive, dangerous and unaesthetic a stigma is perceived, the more individuals report frequent discrimination experiences and higher stigma consciousness. Insights from intergroup research indirectly support this, documenting, for example, that stigmatized groups seen as disruptive to social interactions, unfamiliar, or threatening face stronger prejudice and stereotyping (Gonsalkorale et al., 2007; Grigoryan et al., 2022; Riek et al., 2006). Furthermore, stigmatized group members who believe others view their group as dangerous or the stigma to be under a person's individual control report more frequent discrimination and greater distress (Krendl & Freeman, 2017; Lo et al., 2022; Schwarzer & Weiner, 1991; Weiner et al., 1988). Collectively, prior work highlights perceived stigma controllability, stability, disruptiveness, peril and unattractiveness as associated with more discrimination experiences, which may in turn increase stigma consciousness. However, given the limited systematic research on all six dimensions, we did not preregister directional hypothesis but rather explore these relationships.

#### Group‐level characteristics

Our third theoretical level considers group‐level characteristics that may relate to stigma consciousness. Pinel (1999) postulated that any variable that increases the salience of group membership may increase stigma consciousness. In this line, we propose that (a) societal context, including *status* hierarchies and differences in stigmatization, and (b) group characteristics, including *entitativity* and *permeability* of one's ingroup, affect stigma consciousness.

First, we considered the degree of stigmatization and the significance of its consequences. We assume that stigmatized groups differ in type and degree of stigmatization, which may affect the salience and frequency of stigmatization experiences, and in turn, stigma consciousness. We adopt a sociological definition of stigmatization, which views stigmatization as the joint occurrence of the devaluation and disadvantaging of groups in society (Link & Phelan, 2001). We operationalize group devaluation as relative *sociometric status*—the extent to which a group is (de)valued, (dis)respected or (dis)liked in society—and group disadvantage as *socioeconomic status*—reflecting (lack of) access to financial, educational and occupational resources (Mahadevan et al., 2021). Prior research indicates that lower sociometric and socioeconomic status are indeed linked to more frequent experiences of social exclusion and discrimination (Brondolo et al., 2009; Huebner & Davis, 2007; Jokela & Fuller‐Rowell, 2022). We therefore expected that both status variables correlate negatively with stigma consciousness, in that lower status represents more stigmatization, in turn leading to higher stigma consciousness.

Second, we revisited SIT to identify two further group characteristics that may impact group membership salience and thereby stigma consciousness: group *entitativity* and *permeability*. Entitativity refers to the perception of a group as a unified entity characterized by shared structure, goals and member similarity (Lickel et al., 2000; Yzerbyt et al., 2000). We propose that greater perceived entitativity may increase awareness of the ingroup's stigmatized status, as highly entitative groups are more distinct, making members more attuned to relevant ingroup characteristics, including stigmatization, and thus increasing stigma consciousness. Indeed, perceived entitativity has been shown to be associated with stereotyping, discrimination and prejudice (Agadullina & Lovakov, 2018; Andreychik & Gill, 2014; Hodson & Skorska, 2014; Spencer‐Rodgers et al., 2007), indicating that members of highly entitative groups report more discrimination experiences than those in low entitative groups, indirectly affecting stigma consciousness.

Permeability refers to the extent to which members can leave their ingroup or attain higher individual status (Armenta et al., 2017). Within SIT research, permeability has been shown to moderate how individuals respond to low ingroup status, in that they either distance themselves from the group or identify more strongly with the group (Ellemers et al., 1990). We assume that low permeability may increase members' focus on their ingroup and its defining characteristics, including its stigmatized status, thereby potentially increasing stigma consciousness. Indeed, a study with immigrants in Europe documented that participants who perceived group boundaries as more permeable were less aware of ingroup discrimination (Bobowik et al., 2023). Given the lack of research on the relationship of stigma consciousness and both permeability and entitativity, we did not preregister directional hypotheses but rather explored these relationships.

### The current research

Our primary research question addressed whether, and to what extent, individuals and stigmatized groups differ in stigma consciousness levels, as well as the factors that may predict these differences. We systematically examined individual‐, stigma‐, and group‐level predictors across 18 stigmatized groups in the United States. This approach moves beyond previous research, which has typically focused on a limited number of social groups or stigmatized domains. By encompassing a wide range of groups, we aim to identify patterns that may generalize across stigmatized groups, while also accounting for group‐specific aspects.

At the individual level, we examined discrimination experiences, ingroup identification, internalized stigma, system justification beliefs and ideological orientation as predictors of stigma consciousness. Stigma‐level predictors included six characteristics: concealability, origin, course, disruptiveness, peril and aesthetics. At the group level, we considered socioeconomic and sociometric group status, entitativity and permeability.

Based on the available literature, we preregistered that stigma consciousness would correlate positively with ingroup identification (H1), discrimination experiences (H2), socioeconomic (H3a) and sociometric status (H3b). Given the scarcity of work on the other variables, our inspection of interrelations with all further variables remained explorative. We apply a multilevel approach that allows a clearer understanding of how the proposed predictors of stigma consciousness function at both the individual and group level—thus disentangling within‐group and between‐group processes.

## METHOD

We conducted a cross‐sectional survey with participants from the United States completing an online self‐report questionnaire. All materials and analyses were preregistered in the Open Science Network (Preregistration), which also contains all datasets and analysis scripts (Project). The research question, hypotheses and analyses reported here constitute a subset of a larger project addressing multiple research questions and correspond to hypotheses H1.1 and H1.2 in the preregistration. We only report materials, analyses and results regarding this research goal (see Flöther & Degner, 2025, for investigation of the second research goal). This research was approved by the ethics committee of the psychology department of the University of Hamburg (AZ_2023_024).

### Data collection and sample size determination

Participants were recruited via Prolific based on self‐reported demographic information that indicated membership in one of the included stigmatized groups. Drawing on a sociological conceptualization (Link & Phelan, 2001), we define stigmatization as the joint occurrence of devaluation of a social group (i.e., sociometric status) and limited access to essential resources (i.e., socioeconomic status). Following this, we selected stigmatized groups for this study based on a pilot study, in which a representative sample of *N* = 95 US‐American participants rated the stigmatization level of 31 social groups in US‐American society by rating their socioeconomic and sociometric status (Pilot). We included 18 groups that were rated as low in socioeconomic status and/or sociometric status including people who self‐categorize as African American, Asian American, Native American, Latin American, Alcoholic, wheelchair user, poor people, unemployed people, old people, homosexual, transgender, Muslim, Jewish, Atheists, Conservative, Liberal,^1^ overweight or single parent.

Inclusion criteria required participants to be registered with Prolific as US‐American residents, to be at least 18 years old, to have a minimum of B1‐level English language skills and self‐categorize as belonging to one of the specified stigmatized groups (see Table S1 in Appendix S1 for group‐specific criteria). The survey had an average duration of approximately 19 min and participants were compensated at a rate equivalent to USD $12 per hour. Data were collected sequentially between October 2023 and February 2024^2^ preventing duplicate participation by Prolific users with overlapping group memberships.

Following sample size recommendations for stable estimation of correlations of moderate effect sizes within subsamples (Schönbrodt & Perugini, 2013), we aimed to recruit *n* = 250 participants per stigmatized group. This target sample size ensured high statistical power (1 − *β* = .95) to detect moderate within‐sample correlations of *r*s ≥ .223 and adequate power (1 − *β* = .80) to detect smaller correlations of *r*s ≥ .175 (with *α* = .05, two‐sided). Given the limited number of eligible Prolific users meeting the inclusion criteria for some subsamples (ranging between *n* = 254 and *n* = 9.526 at the time of preregistration), we anticipated that recruitment for certain groups would fall short of the intended target of *n* = 250.

A total of *N* = 5.448 participants were recruited at study onset. As preregistered, we excluded data of participants who did not consent to data storage and analysis (*n* = 2), who did not complete the survey (*n* = 20), whose self‐categorization within our survey was inconsistent with the inclusion criterion for their Prolific invitation (*n* = 1.292), who indicated having less than B1‐level English language skills (*n* = 3), insufficient understanding of items (*n* = 119), who were under 18 years of age (*n* = 1), or who failed the attention check twice (*n* = 42). At measure level, data of *n* = 16 participants who did not respond to >60% of the entitativity items, and *n* = 45 participants who provided identical status ratings for >80% of the target groups in the status rating task, were excluded from analyses involving the respective measures. No additional preregistered exclusion criteria were met. Exclusion of participants per subsample is reported in Tables S2 and S3 in Appendix S1.

### Participants

Analyses presented here are based on data of *N* = 3.969 US‐residents (*n* = 1.998 female [50.34%], *n* = 1.786 male [45.00%], *n* = 27 non‐binary [0.68%], *n* = 9 not listed gender [0.23%], *n* = 149 prefer not to answer [3.75%]) with subsample sizes ranging from *n* = 140 for Native Americans to *n* = 247 for Jews. Participants were between 18 and 86 years of age (*M* = 40.12, *SD* = 14.28). See Table S4 in Appendix S1 for demographics per subsample.

### Measures

#### Stigma consciousness

We adapted nine items from the Stigma Consciousness Questionnaire (SCQ, Pinel, 1999), eight items from the Stigmatization Scale (Harvey, 2001) and seven items from the Anticipated Stigma Scale (Quinn & Chaudoir, 2009) to form a 24‐item scale, measuring awareness and anticipation of ingroup stigmatization on a seven‐point scale (1 = ‘strongly disagree’; 7 = ‘strongly agree’). Due to their similarity and for reasons of test economy, we combined two items of the SCQ (‘My being [ingroup] does not influence how [outgroup] interact with me’; ‘My being [ingroup] does not influence how people interact with me’) into one (‘Being [ingroup] influences how people interact with [ingroup]’).

#### Individual characteristics

##### Discrimination experiences

We measured participants' frequency of experienced discrimination in everyday life with nine items on a five‐point scale (1 = ‘never’; 5 = ‘always’; Williams et al., 1997). Reliability was satisfying across groups with *α* = .93 (.90 ≥ *α* ≥ .95; see Table S5 in Appendix S1).

##### Ingroup identification

We measured the extent to which individuals identified with the ingroup on a seven‐point scale (1 = ‘strongly disagree’; 7 = ‘strongly agree’) with the 14‐item Ingroup Identification Scale (Leach et al., 2008). Reliability was satisfying with Cronbach's *α* = .94 for the overall sample (.80 ≥ *α* ≥ .96 for subsamples; Table S5 in Appendix S1).^3^

##### Internalized stigma

We adapted one item from Puhl et al. (2012), asking participants in an open response format to list five negative stereotypes about their ingroup and indicate to what extent each stereotype applied to them on a seven‐point scale (1 = ‘not at all’, 7 = ‘extremely’). Reliability for the scale was satisfying across groups with *α* = .80 (.64 ≥ *α* ≥ .92; see Table S5 in Appendix S1).

##### System justification believes

We measured perceived fairness and legitimacy of the societal system on a five‐point scale (1 = ‘strongly disagree’; 5 = ‘strongly agree’) using the eight‐item System Justification Scale (Kay & Jost, 2003). Reliability was satisfying with *α* = .88 (.82 ≥ *α* ≥ .90, see Table S5 in Appendix S1).

##### Ideological orientation

We included one item asking participants to ‘indicate where [they] would place [their] political ideology on the following scale’ (1 = ‘extremely liberal’; 7 = ‘extremely conservative’).

#### Stigma characteristics

We adapted six items from Pachankis et al. (2018) to assess participants' perceptions of the general populations' beliefs regarding stigma characteristics (‘To what extent does the general population…’), including its concealability (‘…believe that this identity can be easily concealed in a typical social interaction?’), course (‘…expect this identity or condition to improve or persist, worsen, or recur?’), disruptiveness (‘…disrupt typical social interactions taking place among typical members of the general population, assuming the stigma is known?’), origin (‘…see the stigmatized individual as being responsible for his or her identity or condition?’), aesthetics (‘…does the identity or condition prompt physical revulsion among typical members of the general population in typical social interactions, assuming the stigma is known?’) and peril (‘…perceive some kind of contagion, threat, peril, or physical danger to themselves in typical social interactions, assuming the stigma is known?’). Responses were collected using six‐point scales with item specific verbal anchors on value one (i.e., unconcealable, temporary, non‐disruptive, uncontrollable, not repulsive, not peril) and value six (i.e., concealable, persistent, disruptive, controllable, repulsive, extreme peril).

#### Group characteristics

##### Group status

Perceptions of group status were assessed using two items from Mahadevan et al. (2021), applied to 31 social groups in US–American society, including participants' ingroup. Socioeconomic status was assessed by asking participants to rate each group's financial, educational and occupational resources and opportunities on a seven‐point scale (1 = ‘least money, education, and job’; 7 = ‘most money, education, and job’). Sociometric status was assessed by asking participants to indicate the extent to which a group is disliked, devalued, or disrespected in US‐American society on a seven‐point scale (1 = ‘not at all’; 7 = ‘extremely strongly’). To minimize potential distress or response hesitations due to the negatively worded sociometric status item, participants were (falsely) informed that they had been randomly assigned to either a positive or negative rating condition. The order of the status rating tasks was randomized across participants, and the order of the 31 target groups was randomized within each rating task. For each target group, participants could indicate that they were unfamiliar with a given group, allowing them to skip the rating (coded as missing value). As no systematic missing values were detected across items or subsamples, all target groups were retained in the analyses (Table S3 in Appendix S1). We had preregistered to aggregate both status measures to form an indicator of stigmatization. However, because socioeconomic and sociometric status perceptions were not strongly correlated at the group level, *r*(18) = .322, *p* = .193, or individual level, *r*(3969) = .195, *p* < .001, we treated them as independent variables in our analyses (see Table S6 in Appendix S1 for preregistered analyses).

##### Entitativity

We included 12 items (Rydell & McConnell, 2005) assessing the extent to which participants perceived a collection of people as a unified group on a seven‐point scale (1 = ‘not at all’, 7 = ‘to a large extent’). Given that the items were designed for small groups and might not fully apply to larger categories, participants were provided the option to mark items as not applicable to their ingroup (coded as missing values). No systematic missing values were detected across items or subsamples, and reliability indices were satisfying with *α* = .91 (.80 ≥ *α* ≥ .91, see Tables S3 and S5 in Appendix S1).

##### Permeability

We included 14 items (Armenta et al., 2017) assessing the extent to which participants perceived ingroup boundaries and status as permeable on a five‐point scale (1 = ‘strongly disagree’; 7 = ‘strongly agree’). Reliability indices were satisfying with *α* = .81 (.61 ≥ *α* ≥ .84, see Table S5 in Appendix S1).

##### Demographic data

Participants were asked to indicate their gender, age, ethnicity and English language skills, as well as self‐categorize regarding the inclusion criteria for each subsample (see Table S1 in Appendix S1).

### Procedure

The study was conducted using an online self‐report questionnaire implemented in Qualtrics XM. At the start, participants were informed that the study investigated perceptions of social groups, their characteristics and societal positions, as well as participants' experiences and opinions regarding the ingroup. A trigger warning was included for questions concerning discrimination experiences, along with support resources. Participants were informed about data policies and provided consent for data storage and analysis.

The survey began with measures on group characteristics (socioeconomic and sociometric status, entitativity, permeability), followed by the measure on stigma characteristics (concealability, course, disruptiveness, origin, aesthetics, peril). Subsequently, participants were told which social group had formed the basis of their survey invitation to enhance ingroup salience (‘You were invited to this survey because you indicated to be [group] in Prolific. Please think of this social group identity when answering the following questions’). Participants then completed the measures on individual characteristics (ingroup identification, stigma consciousness, internalized stigma, discrimination experiences, system justification beliefs, ideological orientation). Finally, participants provided demographic information and answered a question about item and instruction comprehensibility.

After completion of all measures, participants were redirected to Prolific to receive payment.

### Data preparation and analysis plan

All statistical analyses were performed using R (version 2022.07.2, R Core Team, 2023, see Appendix S1 for employed R‐packages).

We had employed a stigma consciousness measure consisting of rearranged items of three established stigma measures (Harvey, 2001; Pinel, 1999; Quinn & Chaudoir, 2009), focusing on two subcomponents: stigma awareness and stigma anticipation. However, confirmatory factor analyses (CFA) revealed that this preregistered two‐factorial model did not fit the data well and model fit could not be improved by data‐driven model modifications (see Appendix S2). CFA of the one‐factor structure of the SCQ as originally proposed by Pinel (1999), however, revealed good to acceptable fit for the sample as well as within each subsample. We henceforth included only the SCQ items for analyses, thus deviating from our preregistered plan (Cronbach's *α* = .76; ranging between .66 ≥ *α* ≥ .85 at subsample level).

The result section is structured as follows: First, we provide descriptive statistics of stigma consciousness and all hypothesized predictor variables for the aggregated sample and all subsamples. Second, we report bivariate correlation analyses investigating associations between stigma consciousness and all individual‐, stigma‐, and group‐level predictors on both the sample and subsample level. Third, we extend these analyses by employing multilevel regression modelling to account for the data structure, with individuals nested within groups. Specifically, we estimated random intercept, random slope models to account for potential between‐group variance in stigma consciousness. We began by conducting a null model (random intercept only), which included stigma consciousness as the outcome without predictor variables to determine the intraclass correlation coefficient (*ICC*) as an indicator of variance proportion in stigma consciousness attributable to between‐group differences. We then ran separate multilevel regression models for each predictor to assess their association with stigma consciousness. For individual‐level predictors, we applied group‐mean centring to isolate within‐group effects and included the group mean of the variables to assess corresponding between‐group effects. For group‐level predictors, we deviated from the preregistration by choosing a different centring method. We originally preregistered grand‐mean centring for group‐level predictors to examine overall between‐group effects. However, while we conceptualized group and stigma characteristics as group‐level predictors, these variables were measured at the individual level and can therefore represent both differences in shared perceptions between groups and/or differences in individual perceptions within groups. We thus ran non‐preregistered multilevel regression models including both the group‐mean centred predictor and the group mean to simultaneously distinguish within‐ and between‐group association. Finally, to evaluate the most parsimonious model, we estimated a combined model including all significant individual‐ and group‐level predictors from prior single‐predictor models.

## RESULTS

### Descriptive statistics

Descriptive statistics for all variables for the overall sample are provided in Table 1. Participants reported relatively high levels of stigma consciousness, *M* = 4.54, *SD* = 1.00, ranging between *M* = 5.21, *SD* = 0.83, for transgender people and *M* = 4.00, *SD* = 0.99, for single parents on the subsample level (see Table S7 in Appendix S1).

**TABLE 1:** Descriptive statistics for all variables across groups.

Variables	Mean	*SD*	d*
Stigma consciousness	4.54	1.00	0.54
Individual characteristics			
Ingroup identification	4.73	1.33	0.55
Internalized stigma	2.24	1.24	−1.56
Discrimination experiences	2.01	0.82	−1.20
System justification beliefs	2.42	0.86	−0.67
Ideological orientation	3.12	1.76	−0.50
Stigma characteristics			
Concealability	3.56	1.56	0.04
Course	4.23	1.32	0.55
Disruptiveness	2.73	1.40	−0.55
Origin	4.19	1.45	0.47
Aesthetics	3.08	1.45	−0.29
Peril	2.75	1.49	−0.51
Group characteristics			
Entitativity	3.56	0.85	0.66
Permeability	3.36	0.70	0.52
Socioeconomic status	3.75	1.57	−0.16
Sociometric status	3.21	1.63	−0.48

*Note*: *d = effect size of *t*‐test for deviation from midpoint of the scale (*df* = 3698).

Results of correlation analyses for the overall sample are summarized in Table 2 (see Table S8 in Appendix S1 for subsample analyses). All preregistered hypotheses were supported on the individual level, as stigma consciousness was significantly related to discrimination experiences (H1), ingroup identification (H2), socioeconomic status (H3a) and sociometric status (H3b). Similar significant support was observed at the group level for H1 and H3b, but correlation coefficients were substantially smaller and non‐significant for H2 and H3b.

**TABLE 2:** Intercorrelations of stigma consciousness and predictor variables.

Components		1	2	4	5	6	7	8	9	10	11	12	13	14	15	16	17
	(1) Stigma Consciousness		.113 (<.001)	−.023 (.151)	.415 (<.001)	−.321 (<.001)	−.182 (<.001)	−.217 (<.001)	.065 (<.001)	.259 (<.001)	.096 (<.001)	.368 (<.001)	.296 (<.001)	.066 (<.001)	−.267 (<.001)	−.148 (<.001)	−.361 (<.001)
Individual characteristics	(2) Ingroup identification	−.059 (.816)		−.070 (<.001)	.102 (<.001)	.091 (<.001)	−.003 (.830)	.013 (.408)	.021 (.193)	−.029 (.064)	.031 (.047)	−.031 (.052)	.103 (<.001)	.632 (<.001)	−.198 (<.001)	.366 (<.001)	−.053 (<.001)
	(4) Internalized stigma	−.018 (.944)	−.471 (.049)		.169 (<.001)	.100 (<.001)	.106 (<.001)	.006 (.720)	−.015 (.332)	.095 (<.001)	−.019 (.228)	.055 (<.001)	.008 (.595)	−.031 (.049)	−.117 (<.001)	−.027 (.086)	.080 (<.001)
	(5) Discrimination Experiences	.589 (.010)	.035 (.889)	−.038 (.881)		−.136 (<.001)	.006 (.687)	−.158 (<.001)	−.029 (.065)	.334 (<.001)	−.035 (.026)	.377 (<.001)	.400 (<.001)	.123 (<.001)	−.311 (<.001)	−.160 (<.001)	−.265 (<.001)
	(6) System Justification	−.261 (.295)	.156 (.536)	.373 (.127)	−.218 (.384)		.462 (<.001)	.051 (.001)	.044 (.006)	−.067 (<.001)	.014 (.374)	−.112 (<.001)	−.109 (<.001)	.131 (<.001)	−.018 (.263)	.241 (<.001)	.257 (<.001)
	(7) Ideological Orientation	−.159 (.528)	.061 (.809)	.340 (.168)	−.042 (.867)	.834 (<.001)		−.006 (.721)	−.010 (.534)	−.002 (.904)	.013 (.400)	−.031 (.050)	−.053 (<.001)	.108 (<.001)	−.139 (<.001)	.103 (<.001)	.178 (<.001)
Stigma characteristics	(8) Concealability	−.628 (.005)	.038 (.881)	−.033 (.897)	−.638 (.004)	−.001 (.996)	−.077 (.762)		−.045 (.005)	−.108 (<.001)	.105 (<.001)	−.154 (<.001)	−.097 (<.001)	−.056 (<.001)	.297 (<.001)	.099 (<.001)	.101 (<.001)
	(9) Course	.083 (.744)	.009 (.971)	.198 (.431)	.053 (.836)	.282 (.256)	.052 (.837)	−.280 (.261)		.016 (.317)	.036 (.025)	.043 (.007)	.001 (.963)	.005 (.736)	−.044 (.005)	.048 (.003)	−.010 (.520)
	(10) Disruptiveness	.582 (.011)	−.285 (.252)	.253 (.310)	.760 (<.001)	−.107 (.674)	−.015 (.954)	−.364 (.137)	.057 (.822)		−.007 (.645)	.439 (<.001)	.426 (<.001)	.043 (.007)	−.178 (<.001)	−.162 (<.001)	−.208 (<.001)
	(11) Origin	.156 (.537)	−.167 (.507)	.045 (.860)	−.385 (.115)	−.073 (.772)	−.020 (.938)	.444 (.065)	−.010 (.968)	−.144 (.568)		.096 (<.001)	.029 (.070)	.007 (.642)	.107 (<.001)	.030 (.057)	−.022 (.178)
	(12) Aesthetics	.751 (<.001)	−.289 (.244)	−.004 (.989)	.597 (.009)	−.187 (.458)	−.127 (.615)	−.495 (.037)	.218 (.385)	.684 (.002)	.272 (.275)		.554 (<.001)	.022 (.167)	−.127 (<.001)	−.137 (<.001)	−.352 (<.001)
	(13) Peril	.550 (.018)	.256 (.304)	−.286 (.251)	.706 (.001)	−.188 (.455)	−.138 (.585)	−.216 (.389)	.065 (.798)	.673 (.002)	.082 (.747)	.698 (.001)		.145 (<.001)	−.118 (<.001)	−.097 (<.001)	−.329 (<.001)
Group characteristics	(14) Entitativity	.085 (.738)	.855 (<.001)	−.278 (.264)	.237 (.345)	.295 (.234)	.223 (.374)	−.164 (.515)	.018 (.944)	.029 (.909)	−.307 (.214)	−.129 (.611)	.393 (.107)		−.208 (<.001)	.277 (<.001)	.015 (.345)
	(15) Permeability	−.284 (.253)	−.271 (.276)	−.146 (.564)	−.390 (.110)	−.404 (.096)	−.422 (.081)	.597 (.009)	−.279 (.263)	−.167 (.507)	.520 (.027)	.084 (.739)	−.025 (.920)	−.414 (.088)		.013 (.421)	.007 (.676)
	(16) SES	−.135 (.592)	.621 (.006)	−.034 (.892)	−.411 (.090)	.432 (.074)	.103 (.683)	.140 (.581)	.411 (.090)	−.450 (.049)	.144 (.569)	−.219 (.383)	−.087 (.731)	.432 (.073)	−.061 (.811)		.195 (<.001)
	(17) SMS	−.613 (.007)	.151 (.550)	.308 (.214)	−.576 (.012)	.398 (.102)	.285 (.252)	.249 (.320)	.147 (.561)	−.609 (.007)	−.215 (.391)	−.848 (<.001)	−.771 (<.001)	.017 (.948)	−.332 (.178)	.322 (.193)	

*Note*: Individual‐level correlations (*n* = 3969) above the diagonal, group‐level correlations (*n* = 18) below the diagonal.

Abbreviations: SES, socioeconomic status; SMS, sociometric status.

### Multilevel analyses

#### Stigma consciousness (null model)

We estimated an intercept‐only model to examine the variance in stigma consciousness attributable to differences between subsamples. The model indicated a significant intercept, *b* = 4.54, *SE* = 0.07, *p* < .001, reflecting the average level of stigma consciousness across groups. The estimated between‐group variance was 0.09, and the within‐group variance was 0.91, resulting in an ICC of .092, indicating that only 9.2% of the total variance in participants' stigma consciousness was attributable to differences between subsamples.

For each predictor, we estimated a model separating within‐group and between‐group effects by including group‐mean centred predictor and the group mean of the predictor and stigma consciousness as the outcome variable. Results for each model are summarized in Table 3.

**TABLE 3:** Multilevel regression models with one predictor variable.

	Null model			Intercept variance		Residual variance		ICC
	Estimate (*b*)	*SE*	*p*‐value	Variance	*SD*	Variance	*SD*	
Stigma consciousness	4.54	0.07	<.001	0.09	0.30	0.91	0.96	.092

Model	Individual‐level prediction			Group‐level prediction			Intercept variance		Slope variance		Residual variance		ICC
	Estimate (*b*)	*SE*	*p*‐Value	Estimate (*b*)	*SE*	*p*‐Value	Variance	*SD*	Variance	*SD*	Variance	*SD*	
Individual characteristics													
Ingroup identification	0.16	0.03	<.001	−0.005	0.08	.957	0.10	0.31	0.01	0.11	0.87	0.94	.108
Discrimination experiences	0.43	0.02	<.001	0.59	0.19	.007	0.08	0.28	0.03	0.18	0.71	0.84	.086
Internalized stigma	−0.02	0.01	.073	−0.08	0.21	.700	0.11	0.34	0.04	0.19	0.88	0.94	.115
System justification	−0.41	0.04	<.001	−0.17	0.21	.443	0.09	0.30	0.02	0.14	0.80	0.89	.104
Ideological orientation	−0.13	0.03	<.001	−0.004	0.08	.963	0.10	0.31	0.01	0.10	0.86	0.93	.120
Stigma characteristics													
Concealability	−0.11	0.02	<.001	−0.25	0.06	.001	0.06	0.24	0.003	0.05	0.89	0.94	.062
Aesthetics	0.24	0.02	<.001	0.36	0.08	<.001	0.04	0.20	0.01	0.09	0.81	0.90	.056
Disruptiveness	0.17	0.02	<.001	0.37	0.14	.016	0.06	0.25	0.01	0.08	0.86	0.93	.074
Peril	0.19	0.02	<.001	0.29	0.09	.006	0.07	0.26	0.01	0.08	0.84	0.92	.079
Course	0.05	0.02	.047	0.05	0.23	.827	0.10	0.31	0.01	0.09	0.90	0.95	.109
Origin	0.06	0.02	.002	0.07	0.16	.670	0.10	0.31	0.003	0.06	0.90	0.95	.102
Group characteristics													
Sociometric status	−0.21	0.02	<.001	−0.28	0.09	.006	0.06	0.25	0.004	0.06	0.80	0.89	.069
Socioeconomic status	−0.15	0.02	<.001	−0.04	0.07	.571	0.10	0.31	0.01	0.09	0.87	0.93	.096
Entitativity	0.09	0.04	.018	0.06	0.14	.664	0.10	0.31	0.01	0.11	0.90	0.95	.101
Permeability	−0.44	0.07	<.001	−0.30	0.20	.151	0.09	0.30	0.08	0.29	0.82	0.90	.118

*Note*: Within‐group effects are based on group‐mean centring, between‐group effects on group means. Intercept variance = between‐group variance; residual variance = within‐group variance.

#### Individual‐level predictors

##### Discrimination experiences

Stigma consciousness was significantly associated with both group‐mean centred discrimination experiences, *b* = 0.43, *SE* = 0.02, *p* < .001, and group‐mean discrimination experiences, *b* = 0.59, *SE* = 0.19, *p* = .007. Adding group‐mean discrimination experiences to the model reduced the ICC to .086, indicating that between‐group differences in experienced discrimination explained a substantial proportion of between‐group variance in stigma consciousness. These results suggest that individuals who belong to groups with more frequent discrimination experiences—and who report frequent personal discrimination experiences—exhibit higher stigma consciousness levels.

##### Ingroup identification

Group‐mean centred ingroup identification was significantly associated with stigma consciousness, *b* = 0.16, *SE* = 0.03, *p* < .001, indicating that individuals who identified more strongly with the ingroup scored higher on stigma consciousness. Group‐mean ingroup identification was not significantly related to stigma consciousness, *b* = −0.005, *SE* = 0.08, *p* = .957, suggesting that only individual differences within groups in ingroup identification and not between‐group differences are associated with differences in stigma consciousness.

##### Ideology

For both ideology predictors, within‐group effects but no between‐group effects emerged: Stigma consciousness was significantly predicted by group‐mean centred system justification beliefs, *b* = −0.41, *SE* = 0.04, *p* < .00, but not by group‐mean system justification beliefs, *b* = −0.17, *SE* = 0.21, *p* = .443. Similarly, stigma consciousness was significantly predicted by group‐mean centred ideological orientation, *b* = −0.13, *SE* = 0.03, *p* < .001, but not by group‐mean ideological orientation, *b* = −0.004, *SE* = 0.08, *p* = .963. These results indicate that individuals who perceived the societal system as fair and who expressed less liberal and/or more conservative ideologies scored lower on stigma consciousness, and these effects were independent of group differences in both ideology predictors.

##### Internalized stigma

For internalized stigma, we observed neither significant within‐group effects, *b* = −0.02, *SE* = 0.01, *p* = .073, nor between‐group effects, *b* = −0.08, *SE* = 0.21, *p* = .700, suggesting that internalized stigma was unrelated to stigma consciousness.

#### Stigma characteristics

We observed significant within‐group effects for all group‐mean centred stigma characteristics: concealability, *b* = −0.11, *SE* = 0.02, *p* < .001, course, *b* = 0.05, *SE* = 0.02, *p* = .047, origin, *b* = 0.06, *SE* = 0.02, *p* = .002, disruptiveness, *b* = 0.17, *SE* = 0.02, *p* < .001, aesthetics, *b* = 0.24, *SE* = 0.02, *p* < .001, and peril, *b* = 0.19, *SE* = 0.02, *p* < .001. Individuals who perceived their stigma as more visible, persistent, controllable, disruptive, unappealing, and dangerous tended to express higher stigma consciousness.

We observed significant between‐group effects for group‐mean concealability, *b* = −0.25, *SE* = 0.06, *p* = .001, disruptiveness, *b* = 0.37, *SE* = 0.14, *p* = .016, aesthetics, *b* = 0.36, *SE* = 0.08, *p* < .001, and peril, *b* = 0.29, *SE* = 0.09, *p* = .006. Adding these predictors to their respective model significantly reduced the ICC (between *ICC* = .056 for aesthetics and *ICC* = .079 for peril), indicating that between‐group differences in these stigma characteristics explained some between‐group variance in stigma consciousness. In contrast, group‐mean course, *b* = 0.05, *SE* = 0.23, *p* = .827, and origin, *b* = 0.07, *SE* = 0.16, *p* = .670, were not significantly related to stigma consciousness and did not explain between‐group variance as indicated by *ICC* > .101.

#### Group characteristics

##### Group status

Stigma consciousness was significantly predicted by group‐mean centred sociometric status, *b* = −0.21, *SE* = 0.02, *p* < .001, and group‐mean sociometric status, *b* = −0.28, *SE* = 0.09, *p* = .006. Adding the group‐level predictor to the model decreased the *ICC* to .069, suggesting that sociometric status explained a substantial portion of the between‐group variance in stigma consciousness. These results indicate that individual differences within groups as well as group‐level differences in sociometric status perceptions predicted differences in stigma consciousness.

Analyses of the predictive value of socioeconomic status revealed only significant within‐group effects, *b* = −0.15, *SE* = 0.02, *p* < .001, but no significant effect of group‐mean socioeconomic status, *b* = 0.04, *SE* = 0.07, *p* = .571. Adding the predictor to the model did not substantially reduce the ICC value (*ICC* = .096). These results indicate that individuals who perceived the ingroup's socioeconomic status as lower tended to express higher levels of stigma consciousness, but this effect was independent of between‐group differences in status perceptions.

##### Entitativity

For entitativity, we observed significant within‐group effects: Group‐mean centred entitativity was significantly related to stigma consciousness, *b* = 0.09, *SE* = 0.04, *p* = .018, indicating that higher perceived entitativity was related to higher stigma consciousness. No between‐group effects emerged, *b* = 0.06, *SE* = 0.14, *p* = .664, and adding group‐mean entitativity to the model did not reduce the ICC (*ICC* = .101). The results suggest that while individual perceptions of group entitativity were associated with stigma consciousness, between‐group differences did not account for variance in stigma consciousness.

##### Permeability

Stigma consciousness was significantly predicted by group‐mean centred permeability, *b* = −0.44, *SE* = 0.07, *p* < .001. No significant between‐groups effect emerged, *b* = −0.30, *SE* = 0.20, *p* = .151, and adding the predictor to the model did not substantially reduce the ICC value (*ICC* = .118). The results indicate that the individual perception of group permeability was associated with stigma consciousness, but group differences in permeability perceptions did not account for variance in stigma consciousness.

#### Combined predictors model

Finally, we conducted a multilevel regression model including all predictors that were significantly associated with stigma consciousness in the prior single‐predictor models (Table 4). While most of the individual‐level predictors remained significant, except for peril and entitativity, none of the group‐level predictors were significantly associated with stigma consciousness, indicating that mainly within‐group differences accounted for differences in stigma consciousness.

**TABLE 4:** Combined predictors model.

Predictors	Fixed effects	Estimate (*b*)		*SE*	*t‐*Value	*p*‐Value
	Intercept	5.05		1.72	2.93	.014
Individual level	Discrimination experiences	0.28		0.02	15.30	<.001
	Ingroup identification	0.13		0.01	8.94	<.001
	System justification beliefs	−0.23		0.02	−13.06	<.001
	Ideological orientation	−0.05		0.01	−5.99	<.001
	Concealability	−0.05		0.01	−5.60	<.001
	Aesthetics	0.08		0.01	7.11	<.001
	Disruptiveness	0.03		0.01	2.66	.008
	Course	0.03		0.01	3.36	<.001
	Origin	0.06		0.01	6.20	<.001
	Peril	0.02		0.01	1.54	.125
	Sociometric status	−0.11		0.01	−12.06	<.001
	Socioeconomic status	−0.06		0.01	−4.68	<.001
	Entitativity	0.04		0.02	−1.79	.074
	Permeability	−0.17		0.02	−7.41	<.001
Group level	Discrimination experiences	−0.30		0.46	−0.65	.530
	Concealability	−0.18		0.12	−1.58	.143
	Aesthetics	0.01		0.22	0.47	.650
	Disruptiveness	0.15		0.22	0.65	.527
	Peril	0.10		0.17	0.59	.565
	Sociometric status	−0.08		0.19	−0.40	.697
	**Random effects**	**Variance**	***SD***			
	Intercept variance	0.05	0.21			
	Residual variance	0.59	0.77			
	ICC	0.045				

*Note*: Individual‐level variables were group‐mean centred predictor variables; group‐level variables were group‐mean predictors. Intercept variance = between‐group variance; residual variance = within‐group variance.

As preregistered, we conducted follow‐up analyses with two combined models, including individual‐level predictors in one model and group‐level predictors in a second model. Results of these analyses are reported in Tables S10 and S11 in Appendix S1.

## DISCUSSION

The present research systematically examined the associations of multilevel predictors of stigma consciousness across a diverse range of stigmatized groups, aiming to understand both individual‐ and group‐level variability. Our analyses revealed only minimal variance in stigma consciousness between the investigated stigmatized groups but substantial variability between individuals. In this line, stigma consciousness was consistently and more strongly related to individual‐level predictors, whereas only a subset of group‐level predictors demonstrated significant associations. This pattern even held for variables we had conceptualized as shared among group members, including group status or stigma characteristics.

This pattern strongly suggests that stigma consciousness is best understood as an individual characteristic, shaped primarily by personal perceptions, beliefs and experiences rather than group membership. Our multilevel approach thus provides strong empirical support for Pinel's (1999) original conceptualization. Accordingly, when considering potential antecedents of stigma consciousness, the emphasis should be placed on individual variables. Our findings highlight several promising associations in this regard, which we discuss in more detail below.

While our study enabled us to compare social groups and their members in stigma consciousness levels and the effects of individual and group‐level variables, its cross‐sectional design does not allow us to draw conclusions regarding causal, directional relationships. Although we proposed individual, stigma and group characteristics as predictors of stigma consciousness, these characteristics could just as well be consequences of stigma consciousness—or reciprocally related—and the true strength and direction of these associations remains unresolved. When discussing our findings, we only propose causal directions where we find these theoretically justified, while acknowledging that the reverse or bidirectional processes remain plausible.

### What shapes stigma consciousness?

In the present research, we identified a set of *individual‐level predictors* that accounted for a proportion of the variability in stigma consciousness levels. Among the examined predictors, discrimination experiences, system justification beliefs and ingroup identification emerged as the most influential factors.

Discrimination experiences appeared as the strongest predictor of stigma consciousness, which aligns both with prior theorizing and empirical research (Degner et al., 2021; Molero et al., 2013; Pinel, 1999), highlighting the importance of past experiences for the awareness and anticipation of stigmatization. Discrimination experiences emerged as both an individual‐ and group‐level predictor, suggesting that variability in stigma consciousness reflects not only personal differences but also differences in the extent to which groups are discriminated in society. Pinel (1999) conceptualized discrimination experiences as both antecedent and outcome of stigma consciousness, and our findings do not allow us to determine the directionality of this association: Repeated discrimination may increase expectations of future bias, elevated stigma consciousness may increase the recognition of discrimination, and both processes may operate reciprocally. Longitudinal and experimental research is needed to disentangle the causal pathways and better capture the interplay between stigma consciousness and discrimination experiences.

In addition, individual ideologies were related to stigma consciousness: High stigma conscious participants tended to be more liberal and to perceive the prevailing system as less fair than lower stigma conscious participants. While this result appears, at first glance, to align with System Justification Theory (Jost & Banaji, 1994), the non‐significant correlations between stigma consciousness and stigma internalization do not support the underlying mechanism proposed in SJT: it seems unlikely that stereotype endorsement and internalized inferiority substantially contribute to the formation of stigma consciousness (see Degner et al., 2021; Flöther & Degner, 2026). Rather, it appears more plausible that ideological beliefs more directly relate to the recognition and anticipation of ingroup stigmatization. Similar arguments have been made in prior research, indicating that individual ideologies, including system justification tendencies and political orientation, impact perceptions of groups and social change movements (Brandt & Crawford, 2020). Specifically, research suggests that individuals' ideologies shape intergroup attitudes, perceptions of injustice and evaluations of collective action, with liberals and conservatives differently judging groups and protest causes depending on the extent to which these seem to align with their own ideologies irrespective of ingroup identification (Al‐Amine & Çakmak, 2025; Brandt et al., 2014; Reimer et al., 2025). Extending this work, our findings indicate that ideology may also influence individual differences in the extent to which one is aware of and expects to be stigmatized, further positioning stigma consciousness as a potential influential factor in the relationship between ideology and intergroup dynamics.

Furthermore, individuals who identified more strongly with their ingroup also exhibited higher stigma consciousness. Consistent with Social Identity Theorizing (Tajfel & Turner, 1979), belonging to a social group and the way in which individuals engage with their ingroup impacts their perceptions. Whereas highly identified individuals may reflect more on ingroup status and are more aware of associated stigmatization, less identified individuals might be more likely to distance themselves from the group or misattribute discrimination experiences. Thus, ingroup identification seems to be a more important construct to understand the formation of stigma consciousness than originally proposed by Pinel (1999).

Moreover, while differences between groups in their *stigma characteristics* did not appear to play a central role, individual's subjective perceptions of how their group is viewed by others seemed influential for stigma consciousness: Individuals who believed that the society perceived their group's stigma as less concealable, unaesthetic, dangerous, disruptive, persistent and controllable, exhibited higher stigma consciousness. It thus appears to matter less whether stigmatized conditions (e.g., body weight) are in fact controllable, than individuals' perceptions of what others believe about its controllability.

We find similar patterns for *group characteristics*: Individuals who perceived their group as highly entitative and its boundaries or status as less permeable, reported higher stigma consciousness. This further highlights that subjective perceptions of one's group seem more consequential for stigma consciousness than structural differences in group entitativity or permeability. Prior research finds similar relationships with ingroup identification, indicating that individuals who consider their group membership as changeable, identify less with their ingroup, irrespective of group‐level differences in permeability (Flöther & Degner, 2025). These findings offer a novel perspective, suggesting that while entitativity and permeability are often considered group‐level characteristics (Ellemers et al., 2002; Lickel et al., 2000), they may also reflect individuals' beliefs that are more influential for characteristics like stigma consciousness.

The pattern of results for group status appears more nuanced: sociometric status perception, that is, the perception of the ingroup being devalued or disrespected, impacted stigma consciousness substantially both at the individual and group level. For socioeconomic status, however, only individual‐level differences mattered, and the effect was much smaller. This dissociation supports our decision to treat the status variables as distinct constructs rather than combining them to a single stigmatization measure and is compatible with research in other domains. For example, Mahadevan et al. (2021) found sociometric status to be a stronger and more immediate predictor of self‐esteem than socioeconomic status. The authors hypothesized, that, unlike socioeconomic status, which is often institutionalized and relatively changeable, sociometric status is embedded more implicitly in everyday social interactions and less malleable, potentially exerting a stronger influence on individual perceptions such as stigma consciousness.

To conclude, while broader group context, specifically ingroup status, seems to be of some relevance, our results suggest that personal experiences, perceptions and ideologies are the primary determinants of stigma consciousness, quite firmly positioning stigma consciousness as an individual characteristic. While our cross‐sectional design precludes causal inferences, our analyses highlight which factors are more strongly associated with stigma consciousness, providing a foundation for future research to clarify causal pathways.

### Expanding the framework of antecedents of stigma consciousness

While our analyses highlight several individual‐level predictors of stigma consciousness, a portion of within‐ and between‐group variability remains unexplained, pointing to the need for future research examining additional individual‐level predictors. Pinel (1999) proposed further individual‐level variables that warrant investigation: intergroup contact and socialization messages.

Consistent with SIT theorizing (Tajfel & Turner, 1979), research suggests that frequent, meaningful interactions with outgroup members foster salience of group membership (Paolini et al., 2010). As assumed by Pinel (1999), high salience of group membership may draw attention to one's stigmatized status and thus increase stigma consciousness. Moreover, frequent intergroup contact may increase exposure to discrimination, which—consistent with prior research and our findings—is strongly associated with higher stigma consciousness. Future research should investigate the role of intergroup contact, and whether it affects stigma consciousness by increasing group status salience or by increasing the frequency of discrimination experiences.

Pinel (1999) further speculated that socialization messages by parents, caregivers or educators might impact stigma consciousness. By—deliberately or unintentionally—communicating information, beliefs and values about the stigmatized identity, socialization messages help children navigate their social environments (Hughes et al., 2006; Ruck et al., 2021). However, socialization messages vary in goals and contents, differently impacting psychological outcomes (Hughes et al., 2006; Huguley et al., 2019). For instance, protective socialization, that is, strategies that prepare children for potential discrimination (Hughes et al., 2006; Hughes & Chen, 1997), have been shown to relate to heightened awareness of social structures, discrimination and social injustice (Bañales et al., 2021; Hughes et al., 2006). In contrast, egalitarian socialization emphasizing equality among groups and mainstream cultural values (Hughes et al., 2006; Hughes & Chen, 1997) might relate negatively to stigma consciousness, given the lack of communication about group membership, social status and discrimination. To date, socialization research has mainly focused on Black and African American communities in the United States, leaving the link between socialization messages and stigma consciousness underexplored. Future studies including families from diverse stigmatized groups could clarify if and how these messages shape stigma consciousness.

### Implications of the present findings

The present study focused on correlates of stigma consciousness, motivated by the impact of stigma consciousness on perceptions and experiences of stigmatization, and consequently on a range of psychological outcomes (Bazemore et al., 2010; Pinel & Paulin, 2005; Son & Shelton, 2011). An important direction for future research should be to shift attention back to these outcomes and to examine how the predictors identified here interact, potentially shaping emotional, cognitive and behavioural outcomes of stigmatization. For instance, Nouvilas‐Pallejà et al. (2018) revealed that stigma consciousness affects well‐being and intergroup behaviour through different pathways: While high stigma consciousness was associated with detecting subtle prejudice and in turn greater identity concealment—leading to poorer well‐being—it was also linked to stronger ingroup identification, which fostered collective action and enhanced well‐being. It is therefore important to examine how stigma consciousness interacts with the individual‐level variables identified here, as its impact on psychological outcomes such as concealment or collective action may depend on which pathways are activated or most salient.

In this line, our results may also have indirect practical implications, potentially identifying entry points for effective prevention and interventions to support individuals coping with stigmatization. For example, efforts may be more effective by targeting individual‐level vulnerabilities and resources that enhance resilience, rather than focusing solely on the stigmatized group as a whole.

Lastly, an important direction for future research concerns the generalizability of our findings. While our sample is large and diverse, its scope remains confined to the US context, and the applicability of our findings beyond this setting should be evaluated with caution. Given the substantial differences between countries and cultural contexts regarding distributions of income, education, health systems and status hierarchies, our results may not fully capture the experiences of stigmatized groups more broadly (Solano‐Flores & Nelson‐Barber, 2001). For instance, the legal status and social acceptance of sexual and gender minorities vary considerably across countries, as do the forms and prevalence of racial, ethnic, or religious stigmatization. Such contextual differences may shape not only the frequency and intensity of discrimination experiences but also how stigma is publicly discussed and framed, which in turn may influence stigma consciousness. Future research should therefore examine the extent to which the present findings replicate across diverse cultural and societal contexts.

## CONCLUSION

The present research systematically investigated associations of stigma consciousness and multilevel predictors across diverse stigmatized groups, providing a valuable foundation for future research on antecedents and outcomes of stigma consciousness. Most importantly, the present research contributes to a more precise conceptualization of stigma consciousness as an individual difference variable and highlights a variety of individual‐level predictors as potential antecedents of the variation in stigma consciousness.

## AUTHOR CONTRIBUTIONS

**Joelle‐Cathrin Flöther:** Conceptualization; methodology; data curation; investigation; formal analysis; visualization; writing – original draft; writing – review and editing. **Juliane Degner:** Conceptualization; methodology; funding acquisition; writing – review and editing; supervision.

## FUNDING INFORMATION

This research was supported by funding from the Federal Ministry of Research, Technology and Space (BMFTR) of Germany. the Free and Hanseatic City of Hamburg under the Excellence Strategy of the Federal and State Governments to Juliane Degner and funding from the Deutsche Forschungsgemeinschaft (DFG; German Research Foundation, DE 1728/4‐1).

## CONFLICT OF INTEREST STATEMENT

The authors report there are no competing financial or non‐financial interests to declare.

## Supporting information


Appendix S1.

Appendix S2.

## Data Availability

The present research is part of a larger research project, which was preregistered including hypotheses, methods and analyses with the Open Science Foundation (https://osf.io/8nv3j/), where all materials, methods, datasets and analysis scripts of both the pilot study and the present research are publicly available (see Files *Pilot Study* and *Understanding Stigma Consciousness* in OSF Project).

## Footnotes

^1^
We acknowledge that Conservatives and Liberals may not be prototypical stigmatized groups. However, our pilot data revealed relatively low sociometric status ratings (*M*s = 3.58 and 3.80) with somewhat higher socioeconomic status ratings (*M*s = 4.63 and 4.60), thereby meeting our inclusion criteria. Similar patterns occur for other stigmatized groups, such as Asian Americans and Jewish Americans.

^2^
Note that data collection for the subsamples of Muslims and Jews started on 17th October 2023 and 26th October 2023, respectively, closely after the revived conflict between Hamas and Israel. These events might have had an impact on participants' responding potentially limiting generalizability.

^3^
We deviated from the preregistered plan to include only the centrality subscale and analysed the full scale for comprehensiveness and comparability with prior research. Results were highly similar (Table S9: Appendix S1).
