# Supplementary material for: Understanding stigma consciousness: A multilevel analysis across diverse stigmatized groups
Source: Br J Soc Psychol. 2026 Mar 23;65(2):e70072. doi: 10.1111/bjso.70072 (PMC13009322; doi:10.1111/bjso.70072)
Supplement: Supplementary file 1 — Appendix S1. [file BJSO-65-0-s002.pdf]

Appendix S1

Understanding Stigma Consciousness:
A Multilevel Analysis across Diverse Stigmatized Groups

All statistical analyses were performed using R (version 2022.07.2, R Core Team, 2023) and the following R-packages: *car* (Fox & Weisberg, 2019), *carData* (Fox et al., 2022), *dplyr* (Wickham et al., 2023a), *haven* (Wickham et al., 2023b), *lavaan* (Rosseel, 2012), *lme4* (Bates et al., 2015), *lmtest* (Zeileis & Hothorn, 2002), *performance* (Lüdecke et al., 2021), *psych* (William, 2024), *tidyverse* (Wickham et al., 2019).

Table S.1

Inclusion criteria for each subsample

**Group**	**Invitation to participate based on registered information in Prolific**	**Self-Categorization in the study**
African American	Registered race as Black or African American	Participants indicate ethnicity to be Black or African American
Asian American	Registered race as Asian	Participants indicate ethnicity to be Asian American
Native American	Registered race as Native American or Alaskan Native	Participants indicate ethnicity to be Native American or Alaskan Native
Latin American	Registered race as Latino or Hispanic	Participants indicate ethnicity to be Latino or Hispanic
Alcoholics	Registered health status as previously been in therapy for alcohol use	Participants indicate to be a current or recovering Alcoholic
Poor People	Registered yearly household income ≤ 10.000 USD (excluding students)	Participants are asked to indicate their current employment status and income status. Participants are included if they indicate their income to be poor or low and that they are not currently a student or in education.
Unemployed	Registered employment status as unemployed (excluding students)	Participants indicate their current employment status as unemployed

Overweight people	Registered BMI > 29	Participants indicate their height in feet and inches and their weight in pounds, which is then used to calculate their BMI. Participants are further asked to indicate how they perceive their own weight ("*Thinking about your body weight, which of the following would you say you are?*") on a 7-point scale (1 = "*very underweight*"; 7 = "*very overweight*"). Participants are included if their BMI is >29 and they perceive themselves to be overweight (values of 6 = "*overweight*" or 7 = "*very overweight*")
Wheelchair users	Registered health status as physical disability or reduced mobility	Participants indicate that they use a wheelchair in everyday life due to their health status
Transgender	Registered as transgender	Participants indicate their gender identification to be transgender
Homosexual	Registered sexual orientation as gay or lesbian	Participants indicate their sexual orientation to be homosexual
Conservatives	Registered as conservative on the political spectrum	Participants place themselves as conservative on the political spectrum
Liberals	Registered as liberal on the political spectrum	Participants place themselves as liberal on the political spectrum
Muslims	Registered religious affiliation as Muslim	Participants indicate their religious affiliation to be Islam
Jews	Registered religious affiliation as Jewish	Participants indicate their religious affiliation to be Judaism
Atheists	Registered as Atheist	Participants indicate their religious affiliation to be non-religious (i.e., Atheists)
Old people	Registered as aged 60 years or older	Participants indicate to be 60 years old or older
Single parents	Registered as having children, living with biological child and not living with biological child's parent or spouse/partner	Participants specify their family situation as being a single parent

Note. Invitation to participate for each subsample is based on participant characteristics self-reported in Prolific. To be included in the study, participants had to additionally self-categorize as a member of the stigmatized group that their invitation to participate was based on.

Table S.2

Inclusion of participants across groups and within subsamples

**Sample**	**Data collected (*n*)**	**Participants excluded (*n*)**							**Final dataset (*n*)**
		No consent	Not finished	Underage	Insufficient English skills	Insufficent understanding	Failed attention check	No self-categorization	
All groups	5.448	2	20	1	3	119	42	1.292	3.969
African Americans	253	0	0	0	0	12	6	2	233
Asian Americans	249	0	0	0	0	4	1	3	241
Native Americans	173	0	7	0	0	3	4	19	140
Latin Americans	252	0	0	0	0	6	3	7	236
Alcoholics	390	1	2	0	0	7	3	137	240
Poor people	338	0	0	0	0	4	1	93	240
Unemployed people	263	0	0	0	0	10	4	21	228
Overweight people	338	0	0	0	0	6	0	104	228
Wheelchair users	667	1	5	0	0	3	2	502	154
Transgender	481	0	6	0	0	11	2	266	196
Homosexual	263	0	0	1	0	5	1	23	233
Conservatives	265	0	0	0	0	3	3	29	230
Liberals	251	0	0	0	0	8	0	6	237
Muslims	263	0	0	0	1	9	7	20	226
Jews	271	0	0	0	0	9	2	13	247
Atheists	250	0	0	0	0	3	0	3	244

Old people	258	0	0	0	1	8	1	11	237
Single Parents	223	0	0	0	1	8	2	33	179

Table S.3

Exclusion of participants across groups and within subsample based on preregistered exclusion criteria

	Participant Level					
	Stigma Consciousness	Discrimination Experiences	Identity	Entitativity	Permeability	System Justification
All Groups	0	2	0	16	0	0
African Americans	0	1	0	2	0	0
Asian Americans	0	0	0	0	0	0
Native Americans	0	1	0	0	0	0
Latin Americans	0	0	0	0	0	0
Alcoholics	0	0	0	1	0	0
Poor people	0	0	0	3	0	0
Unemployed people	0	0	0	2	0	0
Overweight people	0	0	0	2	0	0
Wheelchair users	0	0	0	2	0	0
Transgender	0	0	0	1	0	0
Homosexual	0	0	0	0	0	0
Conservatives	0	0	0	0	0	0
Liberals	0	0	0	0	0	0
Muslims	0	0	0	0	0	0

Jews	0	0	0	1	0	0
Atheists	0	0	0	0	0	0
Old people	0	0	0	0	0	0
Single Parents	0	0	0	2	0	0

	**Status ratings**		**Measure Level**
Group	Participants excluded if >80% identical ratings (*n*)	Participants indicating to not know the group (*n*)	Participants indicated measure not applicable to the ingroup (*n*)
All Groups	45	0	0
African Americans	3	0	0
Asian Americans	0	0	0
Native Americans	2	0	0
Latin Americans	3	0	0
Alcoholics	3	0	0
Poor people	3	0	0
Unemployed people	2	0	0
Overweight people	2	0	0
Wheelchair users	3	0	0
Transgender	2	0	0
Homosexual	3	0	0
Conservatives	0	0	0
Liberals	3	0	0
Muslims	0	0	0

Jews	5	0	0
Atheists	4	0	0
Old people	5	0	0
Single Parents	0	0	0

Table S.4

Demographic information per subsample

**Group**	***n***	**Gender *n* (%)**					**Age**			
		**Female**	**Male**	**Non-binary**	**Not listed**	**Prefer not to answer**	***Mean***	***SD***	**Min.**	**Max.**
African Americans	233	110 (47.21)	123 (52.79)	0 (0.00)	0 (0.00)	0 (0.00)	41.85	12.91	20	73
Asian Americans	241	125 (51.87)	112 (46.47)	0 (0.00)	2 (0.83)	2 (0.83)	34.89	10.82	18	68
Native Americans	140	86 (61.42)	45 (32.14)	1 (0.71)	0 (0.00)	8 (5.71)	36.94	11.79	18	67
Latin Americans	236	103 (43.64)	131 (55.50)	0 (0.00)	1 (0.42)	1 (0.42)	33.04	9.35	18	71
Alcoholics	240	88 (36.67)	146 (60.83)	0 (0.00)	0 (0.00)	6 (2.50)	45.53	12.78	19	86
Poor people	240	124 (60.78)	92 (38.33)	1 (0.42)	3 (1.25)	20 (8.33)	38.04	11.74	18	76

Unemployed people	228	127	95	1	0	5	36.14	12.75	18	71
		(55.70)	(41.67)	(0.44)	(0.00)	(2.19)				
Overweight people	228	146	81	0	0	1	41.92	11.89	22	78
		(64.04)	(35.52)	(0.00)	(0.00)	(0.44)				
Wheelchair users	154	81	61	0	1	11	41.70	15.13	19	75
		(52.60)	(39.61)	(0.00)	(0.65)	(7.14)				
Transgender	196	41	75	21	0	59	29.76	9.62	18	78
		(20.92)	(38.27)	(10.71)	(0.00)	(30.10)				
Homosexual	233	81	135	1	0	16	38.63	13.51	18	81
		(34.76)	(57.94)	(0.43)	(0.00)	(6.87)				
Conservatives	230	97	133	0	0	0	45.10	13.37	20	79
		(42.17)	(57.08)	(0.00)	(0.00)	(0.00)				
Liberals	237	146	88	1	0	2	39.50	12.92	18	76
		(65.82)	(37.13)	(0.42)	(0.00)	(0.84)				
Muslims	226	120	104	1	0	1	31.41	11.10	18	72
		(53.10)	(46.02)	(0.44)	(0.00)	(0.44)				
Jews	247	128	103	1	0	15	40.18	16.13	18	77
		(51.82)	(41.70)	(0.40)	(0.00)	(6.07)				
Atheists	244	105	136	0	1	2	37.71	10.72	19	76
		(43.03)	(55.74)	(0.00)	(0.41)	(0.82)				
Old people	237	150	87	0	0	0	66.05	4.82	60	86
		(63.29)	(36.71)	(0.00)	(0.00)	(0.00)				
Single Parents	179	140	39	0	0	0	41.26	10.96	21	70

(78.21)	(21.79)	(0.00)	(0.00)	(0.00)

**Group**	**Ethnicity *n* (%)**							
	n	White/ Caucasian	Black/African American	Asian American	Latino or Hispanic	Native American/ Alaskan Native	Not listed	Prefer not to answer
African Americans	233	0	233	0	0	0	0	0
		(0.00)	(100)	(0.00)	(0.00)	(0.00)	(0.00)	(0.00)
Asian Americans	241	0	0	241	0	0	0	0
		(0.00)	(0.00)	(100)	(0.00)	(0.00)	(0.00)	(0.00)
Native Americans	140	0	0	0	0	140	0	0
		(0.00)	(0.00)	(0.00)	(0.00)	(100)	(0.00)	(0.00)
Latin Americans	236	0	0	0	236	0	0	0
		(0.00)	(0.00)	(0.00)	(100)	(0.00)	(0.00)	(0.00)
Alcoholics	240	159	57	8	10	0	6	0
		(66.25)	(23.75)	(3.33)	(4.17)	(0.00)	(2.50)	(0.00)
Poor people	240	164	30	7	25	3	11	0
		(68.33)	(12.5)	(2.92)	(10.42)	(1.25)	(4.58)	(0.00)
Unemployed people	228	143	23	22	31	0	9	0
		(62.72)	(10.09)	(9.65)	(13.60)	(0.00)	(3.95)	(0.00)
Overweight people	228	181	26	7	9	1	4	0
		(79.39)	(11.40)	(3.07)	(3.95)	(4.39)	(1.75)	(0.00)
Wheelchair users	154	101	23	4	18	2	6	0
		(65.58)	(14.94)	(2.60)	(11.69)	(1.30)	(3.90)	(0.00)
Transgender	196	138	18	10	14	1	15	0

		(70.41)	(9.18)	(5.10)	(7.14)	(0.51)	(7.65)	(0.00)
Homosexual	233	139	38	23	25	0	8	0
		(59.66)	(16.31)	(9.87)	(10.73)	(0.00)	(3.43)	(0.00)
Conservatives	230	176	27	12	12	0	3	0
		(76.52)	(11.74)	(5.22)	(5.22)	(0.00)	(1.30)	(0.00)
Liberals	237	176	30	15	11	1	4	0
		(74.26)	(12.66)	(6.33)	(4.64)	(0.42)	(1.69)	(0.00)
Muslims	226	43	61	89	3	1	29	0
		(19.03)	(26.99)	(39.38)	(1.33)	(0.44)	(12.83)	(0.00)
Jews	247	214	7	1	11	0	14	0
		(86.63)	(2.83)	(0.40)	(4.45)	(0.00)	(5.67)	(0.00)
Atheists	244	195	9	28	8	0	4	0
		(79.92)	(3.69)	(11.48)	(3.28)	(0.00)	(1.64)	(0.00)
Old people	237	172	55	4	4	0	2	0
		(72.57)	(23.21)	(1.69)	(1.69)	(0.00)	(0.84)	(0.00)
Single Parents	179	127	29	4	12	1	6	0
		(70.95)	(16.20)	(2.23)	(6.70)	(0.56)	(3.35)	(0.00)

Table S.5

Reliability of measures

Group	Stigma Consciousness	Entitativity	Permeability	System Justification Tendencies	Ingroup Identification	Identity Centrality	Discrimination Experiences	Internalized Stigma
All groups	.76	.91	.81	.81	.94	.80	.93	.80
African Americans	.80	.88	.61	.87	.94	.83	.93	.91
Asian Americans	.78	.86	.68	.86	.94	.88	.90	.70
Native Americans	.85	.80	.84	.83	.94	.91	.95	.64
Latin Americans	.83	.89	.84	.90	.95	.91	.94	.66
Alcoholics	.66	.89	.82	.87	.91	.78	.94	.88
Poor people	.77	.85	.78	.86	.80	.66	.91	.77
Unemployed	.77	.84	.76	.85	.80	.57	.91	.77
Overweight	.82	.89	.77	.88	.87	.66	.92	.74
Wheelchair	.78	.89	.78	.87	.90	.70	.90	.81
Transgender	.71	.84	.76	.84	.93	.82	.93	.73
Homosexual	.74	.88	.81	.89	.93	.86	.94	.76
Conservatives	.67	.91	.83	.83	.96	.90	.93	.83
Liberals	.66	.88	.80	.88	.95	.92	.92	.80
Muslims	.70	.87	.70	.88	.96	.92	.93	.92
Jews	.77	.90	.83	.90	.92	.87	.94	.64
Atheists	.73	.88	.80	.86	.92	.88	.92	.66
Old people	.75	.86	.73	.82	.91	.81	.91	.83

Single Parents	.77	.90	.82	.87	.86	.81	.90	.79

Table S.6

Testing preregistered preassumptions for combining socioeconomic and sociometric status into a measure of stigmatization

	**Correlation of SES and SMS per subsample**		**Agreement index (r_{wg})**		**Chronbachs Alpha**
**Group**	***r***	***p***	SES	SMS	
African American	.399	<.001	0.46	0.22	.56
Asian American	-.009	.884	0.77	0.55	-.02
Native American	.329	<.001	0.64	0.27	.47
Latin American	.337	<.001	0.73	0.48	.48
Alcoholics	.129	.047	0.59	0.43	.23
Poor People	-.004	.946	0.87	0.39	-.01
Unemployed people	.152	.022	0.73	0.41	.25
Overweight people	.099	.138	0.82	0.59	.17
Wheelchair users	.281	<.001	0.72	0.40	.42
Transgender	.313	<.001	0.63	0.72	.47
Homosexual people	.055	.409	0.69	0.49	.10
Conservatives	.090	.181	0.75	0.46	.15
Liberals	.189	.004	0.78	0.63	.31
Muslims	.133	.046	0.59	0.20	.22
Jews	-.120	.062	0.75	0.47	-.25
Atheists	-.073	.262	0.81	0.38	-.13
Old people	.157	.017	0.54	0.32	.27
Single parents	.076	.312	0.75	0.39	.13
**Aggregated data**	**Correlation of SMS and SES**		**Chronbachs Alpha**		
Sample level	*r*(18) = .322, *p* = .193		Alpha(18) = .44		
Subsample level	*r*(3969) = .195, *p* <.001		Alpha(3969) = .33		

Note. SES = Socioeconomic status; SMS = Sociometric status.

Table S.7

Descriptive Statistics for stigma consciousness and predictor variables per subsample

Group	*Stigma Consciousness*			*Socioeconomic Status*			*Sociometric Status*			*Entitativity*			*Permeability*		
	M	*SD*	*d*	*M*	*SD*	*d*	*M*	*SD*	*d*	*M*	*SD*	*d*	*M*	*SD*	*d*
African American	4.95	1.09	0.87	3.98	1.47	-0.01	2.80	1.77	-0.68	4.11	0.67	1.66	2.74	0.51	-0.50
Asian American	4.54	0.93	0.58	5.29	0.95	1.35	4.24	1.35	0.18	3.74	0.66	1.11	2.79	0.49	-0.42
Native American	4.35	1.23	0.28	2.57	1.20	-1.20	3.42	1.71	-0.34	4.08	0.50	2.15	3.21	0.74	0.28
Latin American	4.32	1.08	0.29	3.36	1.03	-0.62	3.55	1.44	-0.31	3.95	0.61	1.57	3.26	0.69	0.38
Alcoholics	4.48	0.88	0.55	3.02	1.29	-0.76	2.66	1.51	-0.88	3.45	0.79	0.56	3.56	0.63	0.90
Poor People	4.71	0.96	0.74	1.39	0.73	-3.58	2.75	1.56	-0.80	3.07	0.69	0.11	3.18	0.60	0.30
Unemployed people	4.42	0.98	0.43	1.96	1.04	-1.96	2.97	1.54	-0.67	2.59	0.66	-0.61	3.87	0.55	1.58
Overweight people	5.02	1.05	0.97	3.51	0.86	-0.58	2.49	1.28	-1.18	2.78	0.80	-0.28	3.60	0.59	1.02
Wheelchair users	4.71	0.98	0.73	2.94	1.06	-1.00	3.28	1.56	-0.46	3.13	0.77	0.17	2.92	0.63	-0.13
Transgender	5.21	0.83	1.45	2.90	1.22	-0.90	1.75	1.05	-2.13	3.77	0.57	1.36	3.60	0.58	1.02
Homosexual people	4.44	0.93	0.48	4.32	1.12	0.29	2.73	1.43	-0.89	3.84	0.65	1.28	3.66	0.65	1.02
Conservatives	4.50	0.87	0.58	4.87	1.00	0.87	3.76	1.47	-0.16	3.98	0.66	1.49	3.11	0.64	0.18
Liberals	4.80	0.85	0.94	4.75	0.94	0.80	3.93	1.22	-0.06	3.80	0.60	1.33	3.29	0.59	0.49
Muslims	4.48	0.92	0.52	4.48	1.27	0.37	2.90	1.79	-0.61	4.11	0.63	1.77	3.42	0.57	0.73
Jews	4.33	0.95	0.34	5.48	1.00	1.47	2.78	1.45	-0.84	4.04	0.63	1.64	3.83	0.62	1.34
Atheists	4.09	0.90	0.09	4.63	0.88	0.71	3.75	1.57	-0.16	2.57	0.72	-0.60	3.86	0.55	1.56
Old people	4.42	0.82	0.51	3.89	1.36	-0.08	3.77	1.65	-0.14	3.74	0.65	1.13	2.94	0.53	-0.12
Single parents	4.00	0.99	<0.01	2.99	1.01	-1.00	4.31	1.56	0.20	3.29	0.80	0.36	3.57	0.64	0.90

Stigma Characteristics	*Concealability*			*Course*			*Disruptiveness*			*Origin*			*Aesthetics*			*Peril*		
	M	*SD*	*d*	*M*	*SD*	*d*	*M*	*SD*	*d*	*M*	*SD*	*d*	*M*	*SD*	*d*	*M*	*SD*	*d*
African American	2.89	1.64	-0.37	4.20	1.37	0.51	3.02	1.43	-0.34	4.15	1.50	0.43	3.55	1.38	0.03	3.54	1.50	0.03

Asian American	2.72	1.27	-0.62	4.39	1.12	0.79	2.33	1.09	-1.07	3.67	1.40	0.12	2.50	1.14	-0.88	2.35	1.14	-1.01
Native American	3.32	1.24	-0.14	3.65	1.39	0.11	2.46	1.38	-0.75	3.63	1.58	0.09	2.30	1.28	-0.93	2.61	1.45	-0.61
Latin American	3.31	1.18	-0.17	4.24	1.14	0.65	2.45	1.24	-0.85	3.67	1.41	0.12	2.56	1.25	-0.76	2.80	1.30	-0.54
Alcoholics	3.97	1.24	0.38	4.39	1.25	0.71	3.81	1.27	0.25	4.18	1.35	0.50	3.82	1.26	0.25	3.74	1.38	0.17
Poor People	3.55	1.23	0.04	4.43	1.29	0.72	3.18	1.40	-0.23	4.32	1.40	0.58	3.48	1.35	-.01	3.23	1.46	-0.18
Unemployed people	4.51	1.36	0.74	3.33	1.57	-0.11	2.61	1.39	-0.64	4.32	1.40	0.58	2.75	1.49	-0.50	1.88	1.14	-1.43
Overweight people	1.89	1.13	-1.42	4.60	1.18	0.93	2.64	1.38	-0.62	4.89	1.28	1.09	4.47	1.13	0.86	2.55	1.50	-0.63
Wheelchair users	1.93	1.23	-1.28	4.54	1.38	0.75	3.31	1.42	-0.13	3.05	1.45	-0.31	3.25	1.44	-0.18	2.38	1.41	-0.80
Transgender	2.96	1.43	-0.38	3.69	1.46	0.13	3.46	1.47	-0.03	4.46	1.36	0.71	4.25	1.25	0.60	4.09	1.31	0.45
Homosexual people	3.82	1.28	0.25	4.44	1.25	0.75	2.85	1.38	-0.47	4.06	1.41	0.40	3.52	1.24	0.01	3.27	1.34	-0.17
Conservatives	4.08	1.28	0.45	4.19	1.27	0.54	2.67	1.41	-0.59	4.65	1.20	0.96	2.82	1.35	-0.51	2.53	1.34	-0.72
Liberals	4.16	1.29	0.51	4.43	1.14	0.82	2.61	1.27	-0.70	4.55	1.19	0.89	2.45	1.25	-0.84	2.27	1.24	-0.99
Muslims	3.42	1.33	-0.06	4.33	1.21	0.69	2.61	1.40	-0.64	4.28	1.38	0.57	3.20	1.34	-0.23	3.31	1.48	-0.13
Jews	4.25	1.26	0.60	4.26	1.21	0.62	2.29	1.26	-0.96	4.09	1.35	0.44	2.77	1.31	-0.55	2.72	1.46	-0.54
Atheists	5.40	1.09	1.74	4.34	1.30	0.64	1.97	1.19	-1.29	4.88	1.47	0.94	2.56	1.34	-0.70	2.24	1.29	-0.98
Old people	2.77	1.30	-0.56	4.34	1.37	0.61	2.61	1.29	-0.69	3.84	1.57	0.21	2.91	1.37	-0.43	2.09	1.27	-1.11
Single parents	4.41	1.37	0.66	4.07	1.26	0.45	2.38	1.32	-0.85	4.16	1.29	0.51	2.04	1.25	-1.17	1.60	1.04	-1.82
Individual Characteristics	***Ingroup Identification***			***Identity Centrality***			***Discrimination Experiences***			***Internalized Stigma***			***System Justification Beliefs***			***Ideological Orientation***		
	M	*SD*	*d*	*M*	*SD*	*d*	*M*	*SD*	*d*	*M*	*SD*	*d*	*M*	*SD*	*d*	*M*	*SD*	*d*
African American	5.79	0.99	1.81	5.75	1.28	1.37	2.49	0.87	-0.59	1.96	1.45	-1.40	2.74	0.87	-0.30	3.60	1.86	-0.22
Asian American	5.24	1.01	1.24	5.17	1.34	0.88	1.97	0.62	-1.66	2.46	1.16	-1.33	2.58	0.76	-0.56	3.18	1.24	-0.66
Native American	5.43	1.06	1.34	5.40	1.57	0.90	2.32	0.93	-0.73	1.83	0.93	-2.32	2.05	0.66	-1.45	3.01	1.47	-0.67

Latin American	5.37	1.06	1.28	5.15	1.49	0.77	1.96	0.81	-1.29	1.57	0.90	-2.71	2.48	0.88	-0.59	3.16	1.57	-0.53
Alcoholics	3.66	1.16	-0.29	3.56	1.53	-0.29	2.40	0.94	-0.64	2.79	1.51	-0.80	2.67	0.85	-0.39	3.37	1.92	-0.33
Poor People	3.49	0.77	-0.67	3.56	1.32	-0.33	2.27	0.83	-0.89	2.00	1.10	-1.81	2.09	0.76	-1.20	3.13	1.72	-0.51
Unemployed people	3.06	0.81	-1.16	3.42	1.18	-0.49	1.71	0.70	-1.85	2.12	1.16	-1.61	2.32	0.77	-0.89	3.04	1.54	-0.62
Overweight people	3.30	0.95	-0.74	3.69	1.35	-0.23	2.10	0.75	-1.21	2.34	1.14	-1.46	2.28	0.84	-0.86	3.00	1.66	-0.60
Wheelchair users	4.09	1.05	0.09	4.38	1.44	0.26	2.50	0.76	-0.65	2.21	1.27	-1.41	2.32	0.83	-0.82	3.05	1.74	-0.54
Transgender	5.19	1.07	1.11	5.56	1.24	1.25	2.40	0.83	-0.72	1.68	1.07	-2.17	1.70	0.63	-2.06	1.82	1.36	-1.61
Homosexual people	5.24	1.08	1.15	5.14	1.57	0.72	2.04	0.85	-1.13	1.79	1.02	-2.16	2.27	0.84	-0.87	2.12	1.37	-1.38
Conservatives	5.30	1.10	1.18	4.70	1.58	0.44	1.76	0.75	-1.67	2.42	1.58	-1.00	3.17	0.78	0.22	5.86	0.78	2.40
Liberals	5.13	0.99	1.14	4.55	1.51	0.37	1.72	0.66	-1.94	2.21	1.29	-1.38	2.19	0.77	-1.05	1.97	0.88	-2.29
Muslims	5.90	1.10	1.72	5.96	1.39	1.41	2.20	0.84	-0.95	1.58	1.19	-2.03	2.54	0.83	-0.56	3.68	1.55	-0.21
Jews	5.52	0.86	1.77	5.56	1.24	1.26	1.75	0.75	-1.68	1.86	0.89	-2.41	2.47	0.87	-0.60	2.75	1.52	-0.82
Atheists	4.58	1.11	0.52	3.95	1.79	-0.03	1.51	0.61	-2.43	1.77	0.95	-2.35	2.20	0.75	-1.07	2.27	1.30	-1.33
Old people	4.44	1.06	0.42	4.19	1.50	0.13	1.75	0.67	-1.88	2.01	1.10	-1.81	2.89	0.73	-0.15	3.76	1.79	-0.13
Single parents	4.54	0.91	0.59	4.31	1.42	0.22	1.73	0.65	-1.97	2.54	1.42	-1.03	2.39	0.80	-0.76	3.26	1.66	-0.44

Note.

Table S.8

Correlations of stigma consciousness with group characteristics per subsample

Stigma Consciousness	Entitativity		Permeability		Socioeconomic Status		Sociometric Status	
	r	*p*	*r*	*p*	*r*	*p*	*r*	*p*
African Americans	-.037	.574	-.433	<.001	-.379	<.001	-.470	<.001
Asian Americans	.058	.367	-.441	<.001	-.024	.715	-.363	<.001

Native Americans	.227	.007	-.534	<.001	-.229	.007	-.521	<.001
Latin Americans	.195	.003	-.470	<.001	-.339	<.001	-.482	<.001
Alcoholics	.124	.057	.132	.040	-.183	.005	-.267	<.001
Poor	-.076	.246	-.295	<.001	-.138	.034	-.345	<.001
Unemployed	.068	.311	-.208	.002	-.202	.002	-.346	<.001
Overweight	.051	.445	-.338	<.001	-.146	.029	-.307	<.001
Wheelchair	-.047	.562	-.199	.013	-.286	<.001	-.277	<.001
Transgender	.104	.148	-.177	.013	-.370	<.001	-.316	<.001
Homosexual	.132	.044	-.258	<.001	-.129	.052	-.244	<.001
Conservatives	.146	.026	-.170	.010	-.079	.236	-.182	.006
Liberals	.043	.513	-.163	.012	-.091	.165	-.158	.016
Muslims	-.045	.498	.084	.208	-.029	.668	-.332	<.001
Jews	.082	.200	-.299	<.001	-.059	.361	-.258	<.001
Atheists	.232	<.001	-.260	<.001	.027	.679	-.453	<.001
Old people	-.113	.083	-.357	<.001	-.132	.045	-.328	<.001
Single Parents	.044	.562	-.395	<.001	-.198	.008	-.252	<.001

**Stigma Consciousness**	**Concealability**		**Course**		**Disruptiveness**		**Origin**		**Aesthetics**		**Peril**	
	r	*p*	*r*	*p*	*r*	*p*	*r*	*p*	*r*	*p*	*r*	*p*
African Americans	-.119	.070	.229	<.001	.111	.093	.022	.738	.274	<.001	.392	<.001
Asian Americans	-.216	<.001	.169	.009	.146	.024	-.035	.584	.221	<.001	.199	.002
Native Americans	-.324	<.001	-.116	.173	.471	<.001	-.007	.938	.512	<.001	.468	<.001
Latin Americans	-.113	.082	.140	.032	.278	<.001	.134	.040	.360	<.001	.374	<.001
Alcoholics	-.049	.450	.210	.001	.263	<.001	.050	.441	.316	<.001	.199	.002
Poor	-.121	.062	.158	.014	.272	<.001	.321	<.001	.346	<.001	.330	<.001
Unemployed	-.038	.571	.053	.428	.294	<.001	.305	<.001	.285	<.001	.136	.040
Overweight	-.234	<.001	.096	.147	.332	<.001	.122	.066	.522	<.001	.238	<.001
Wheelchair	-.127	.116	.049	.544	.205	.011	.024	.768	.403	<.001	.235	.003
Transgender	-.241	<.001	-.206	.004	.243	<.001	.165	.021	.384	<.001	.304	<.001
Homosexual	-.030	.653	-.131	.046	.098	.134	.080	.222	.295	<.001	.300	<.001
Conservatives	-.008	.906	.121	.066	.113	.087	.142	.032	.130	.050	.083	.208
Liberals	-.118	.070	.153	.018	.154	.018	.129	.047	.203	.002	.162	.012
Muslims	-.109	.101	.193	.004	.158	.017	.005	.940	.194	.003	.331	<.001
Jews	-.276	<.001	-.022	.729	.346	<.001	.131	.039	.415	<.001	.364	<.001

Atheists	-.063	.324	-.139	.030	.267	<.001	.025	.698	.440	<.001	.389	<.001
Old people	-.226	<.001	.012	.860	.066	.313	-.075	.248	.337	<.001	-.008	.905
Single Parents	-.201	.007	.167	.025	.318	<.001	.077	.310	.144	.056	.130	.083

**Stigma Consciousness**	**Identity**		**Identity Centrality**		**Discrimination Experiences**		**Internalized Stigma**		**System Justification Beliefs**		**Ideological Orientation**	
	r	*p*	*r*	*p*	*r*	*p*	*r*	*p*	*r*	*p*	*r*	*p*
African Americans	.171	.009	.314	<.001	.290	<.001	-.189	.004	-.496	<.001	-.341	<.001
Asian Americans	.110	.088	.264	<.001	.449	<.001	-.085	.191	-.487	<.001	-.290	<.001
Native Americans	.356	<.001	.514	<.001	.700	<.001	-.076	.374	-.301	<.001	-.417	<.001
Latin Americans	.287	<.001	.390	<.001	.557	<.001	.059	.369	-.459	<.001	-.285	<.001
Alcoholics	.160	.013	.209	.001	.161	.013	-.107	.099	-.302	<.001	-.169	.009
Poor	.231	<.001	.456	<.001	.494	<.001	.097	.136	-.341	<.001	-.289	<.001
Unemployed	.118	.076	.274	<.001	.360	<.001	.001	.987	-.283	<.001	-.302	<.001
Overweight	.119	.073	.323	<.001	.488	<.001	.099	.135	-.234	<.001	-.196	.003
Wheelchair	.015	.856	.210	.009	.456	<.001	.018	.824	-.480	<.001	-.123	.129
Transgender	.109	.129	.259	<.001	.404	<.001	-.042	.557	-.469	<.001	-.357	<.001
Homosexual	.249	<.001	.329	<.001	.413	<.001	.042	.522	-.275	<.001	-.197	.003
Conservatives	.300	<.001	.273	<.001	.161	.014	-.155	.019	-.128	.052	.177	.007
Liberals	.161	.013	.280	<.001	.279	<.001	.136	.037	-.353	<.001	-.255	<.001
Muslims	.071	.287	.151	.024	.361	<.001	-.126	.059	-.444	<.001	-.234	<.001
Jews	.193	.002	.331	<.001	.386	<.001	-.121	.057	-.067	.297	.069	.280
Atheists	.403	<.001	.449	<.001	.513	<.001	.022	.731	-.244	<.001	-.228	<.001
Old people	-.149	.022	.101	.120	.271	<.001	.010	.880	-.369	<.001	.008	.900
Single Parents	.112	.137	.465	<.001	.407	<.001	.030	.695	-.318	<.001	-.045	.554

Note. Values represent *Pearson* bivariate correlations. Entries are reported as *r*(*p*). SES = Socioeconomic Status; SMS = Sociometric Status; SJT = System Justification Tendencies.

Stigma Consciousness and Identity Centrality

We originally preregistered to use only the identity centrality subscale of the multicomponent ingroup identification scale (Leach et al., 2008) in our analyses. However, we deviated from the preregistration and analyzed the full scale to capture the relationship of stigma consciousness

and all components of ingroup identification and ensure comparability with prior research. Reliability for the centrality subscale was satisfactory, with $\alpha = .80$ ($.57 \geq \alpha \geq .92$, see Table S.5).

Overall, individuals reported relatively high levels of identity centrality ($M = 4.65$, $SD = 1.65$; see Table S.8 for descriptive statistics at subsample level). Correlations analyses of identity centrality and stigma consciousness as well as the included predictor variables are provided in Table S.9.

Multilevel regression analyses revealed that stigma consciousness was significantly predicted by group-mean centered identity centrality, $b = 0.21$, $SE = 0.02$, $p < .001$, but not by group mean identity centrality, $b = 0.05$, $SE = 0.09$, $p = .612$, indicating that individuals for whom ingroup membership was more central to their self-concept also exhibited higher levels of stigma consciousness, whereas group-level differences in identity centrality did not predict differences in stigma consciousness.

Table S.9

Correlations of Identity Centrality and all other variables included

	Group level correlations (n = 18)	Individual level correlations (n = 3969)
Stigma Consciousness	.122<br>(.629)	.275<br>(<.001)
Individual Characteristics		
Ingroup Identification	.934<br>(<.001)	.814<br>(<.001)
Internalized stigma	-.541<br>(.020)	-.023<br>(.142)
Discrimination Experiences	.256<br>(.305)	.202<br>(<.001)
System Justification Beliefs	.010<br>(.970)	.015<br>(.332)
Political Orientation	-.026<br>(.919)	-.045<br>(.005)
Stigma Characteristics		
Concealability	-.177	.075

	(.483)	(<.001)
Course	-.091	.022
	(.720)	(.172)
Disruptiveness	-.147	.081
	(.561)	(<.001)
Origin	-.267	.007
	(.283)	(.656)
Aesthetics	-.091	.096
	(.720)	(<.001)
Peril	.384	.155
	(.116)	(<.001)
Group Characteristics		
Entitativity	.837	.456
	(<.001)	(<.001)
Permeability	-.249	-.231
	(.319)	(<.001)
Socioeconomic Status	.472	.221
	(.048)	(<.001)
Sociometric Status	-.097	-.060
	(.703)	(<.001)

Multiple Predictors Models

Individual-level Model. We conducted a multilevel regression model including all individual-level predictors simultaneously (Table S.10). All group-mean centered predictors except for peril and entitativity were significantly associated with stigma consciousness, indicating that differences within the groups (i.e., individual differences between participants) in these variables explained differences in stigma consciousness levels.

Table S.10

Individual Level predictors Model

**Fixed Effects**	**Estimate (*b*)**	***SE***	***t*-value**	***p*-value**
Intercept	4.54	0.07	62.18	<.001
**Individual Characteristics**				
Discrimination Experiences	0.29	0.02	15.85	<.001
Ingroup Identification	-0.13	0.01	8.84	<.001
Internalized Stigma	-0.05	0.01	-4.46	<.001
System Justification Beliefs	0.22	0.02	-12.81	<.001
Ideological Orientation	0.05	0.01	-5.89	<.001
**Stigma Characteristics**				
Concealability	-0.05	0.01	-5.52	<.001
Aesthetics	0.08	0.01	7.19	<.001
Disruptiveness	0.03	0.01	2.80	.005
Peril	0.02	0.01	1.45	.149
Course	0.03	0.01	3.32	<.001
Origin	0.05	0.01	6.08	<.001
**Group Characteristics**				
Sociometric Status	-0.10	0.01	-11.71	<.001
Socioeconomic Status	-0.05	0.01	-4.45	<.001
Entitativity	0.04	0.02	-1.83	.068
Permeability	-0.17	0.02	-7.71	<.001

Random Effects	Variance	*SD*
Intercept variance	0.09	0.31
Residual variance	0.58	0.76

Note. Group-mean centered predictor variables were used for this model. Intercept variance = between-group variance; residual variance = within-group variance.

Group-level Model. We additionally conducted a multilevel regression model including all group level predictors simultaneously (Table S.11). We used grand-mean centering for predictor variables to reduce potential multicollinearity and enhance interpretability of results.

Table S.11

Group Level predictors Model

Fixed Effects	Estimate (*b*)	*SE*	*t*-value	*p*-value
Intercept	4.54	0.03	146.78	<.001
**Individual Characteristics**				
Discrimination Experiences	-0.36	0.54	-0.67	.567
Ingroup Identification	-0.10	0.25	0.39	.729
Internalized Stigma	0.10	0.30	0.33	.771
System Justification Beliefs	-0.08	0.38	-0.22	.844
Ideological Orientation	-0.08	0.10	-0.76	.525
**Stigma Characteristics**				
Concealability	-0.35	0.22	-1.54	.260

Aesthetics	-0.55	0.49	-1.13	.373
Disruptiveness	0.54	0.27	2.01	.174
Peril	-0.02	0.24	-0.09	.936
Course	-0.04	0.20	-0.19	.864
Origin	0.56	0.23	2.41	.138
**Group Characteristics**				
Sociometric Status	-0.56	0.32	-1.74	.221
Socioeconomic Status	0.07	0.12	0.61	.598
Entitativity	-0.22	0.33	-0.68	.563
Permeability	-0.53	0.26	-2.03	.181
**Random Effects**	**Variance**	***SD***		
Intercept variance	0.01	0.11		
Residual variance	0.91	0.96		

Note. Grand mean centered predictor variables were used for this model to reduce collinearity. Intercept variance = between-group variance; residual variance = within-group variance.

References

Bates, D., Mächler, M., Bolker, B., & Walker, S. (2015). Fitting linear mixed-effects models using lme4. *Journal of Statistical Software, 67*(1), 1-48. https://doi.org/10.18637/jss.v067.i01

Fox, J., & Weisberg, S. (2019). *An R Companion to applied regression*, (3rd edition). Sage publications. https://www.john-fox.ca/Companion/

Fox, J., Weisberg, S., & Price, B. (2022). CarData: Companion to applied regression data sets. *CRAN: Contributed packages*, https://r-forge.r-project.org/projects/car

Lüdecke, D., Ben-Shachar, M., Patil, I., Waggonner, P., & Makowski, D. (2021). Performance: An R Package for assessment, comparison and testing of statistical models. *Journal of Open Source Software*, 6(60), 3139. https://doi.org/10.21105/joss.03139

Rosseel, Y. (2012). Lavaan: An R package for structural equation modeling. Journal of Statistical Software, 48(2), 1-36. https://doi.org/10.18637/jss.v048.i02

Wickham, H., Averick, M., Bryan, J., Chang, W., McGowan, L. D., François, R., Grolemund, G., Hayes, A., Henry, L., Hester, J., Kuhn, M., Pedersen, T. L., Miller, E., Bache, S. M., Müller, K., Ooms, J., Robinson, D., Seidel, D. P., Spinu, V., Takahashi, K., Vaughan, D., Wilke, C., Woo, K., & Yutani, H. (2019). Welcome to tidyverse. *Journal of Open Source Software, 4*(43), 1686. https://doi.org/10.21105/joss.01686

Wickham, H., François, R., Henry, L., Müller, K., & Vaughan, D. (2023a). Dplyr: A grammar of data manipulation. *CRAN: contributed packages*, https://doi.org/10.32614/CRAN.package.dplyr

Wickham, H., Miller, E., & Smith, D. (2023b). Haven: Import and export 'SPSS', 'Stata' and 'SAS' Files. *CRAN: contributed packages*, https://doi.org/10.32614/CRAN.package.haven

William, R. (2024). Psych: Procedures for psychological, psychometric, and personality research. *CRAN: contributed packages*, https://doi.org/10.32614/CRAN.package.psych

Zeileis, A., & Hothorn, T. (2002). Diagnostic checking in regression relationships. *R News, 2*(3), 7-10. https://cran.r-project.org/doc/Rnews/
