# Supplementary material for: Understanding stigma consciousness: A multilevel analysis across diverse stigmatized groups
Source: Br J Soc Psychol. 2026 Mar 23;65(2):e70072. doi: 10.1111/bjso.70072 (PMC13009322; doi:10.1111/bjso.70072)
Supplement: Supplementary file 2 — Appendix S2. [file BJSO-65-0-s001.pdf]

Appendix S2

Understanding Stigma Consciousness:
A Multilevel Analysis across Diverse Stigmatized Groups

Overview of Appendix S2

This document titled “Appendix S2” contains additional information and supplemental material on the analysis of the factor structure of the stigma consciousness measure. We employed a measure of stigma consciousness of rearranged items of three established stigmatization measures (Harvey, 2001; Pinel, 1999; Quinn & Chaudoir, 2009) and hypothesized a two-factor structure differentiating the dimension stigma consciousness into its components stigma awareness and stigma anticipation. In the following, we report our analysis of the factor structure as well as post-hoc modifications and analyses in detail.

All statistical analyses were performed using R (version 2022.07.2, R Core Team, 2023) and the following R-packages: *car* (Fox & Weibserg, 2019), *carData* (Fox et al., 2022), *dplyr* (Wickham et al., 2023a), *GPArotation* (Bernaards & Jennrich, 2005), *haven* (Wickham et al., 2023b), *lavaan* (Rosseel, 2012), *psych* (William, 2024), *MVN* (Korkmaz et al., 2014), *Rfast* (Papadakis et al., 2023), *tidyverse* (Wickham et al., 2019).

Results of the analysis of the factor structure of stigma consciousness

Descriptive Statistics

Descriptive statistics for the sample (indicated as *all groups*) as well as each subsample including means, standard deviations and reliability and correlations between the two factors can be found in Table S.1.

Table S.1

Descriptive statistics of stigma consciousness variables

Group	Stigma Consciousness			Stigma Awareness			Stigma Anticipation			Correlation of Subscales
	M	*SD*	α	*M*	*SD*	α	*M*	*SD*	α	*r(p)*
All groups	4.14	1.03	.93	4.90	1.06	.89	3.25	1.32	.91	.549 (<.001)

African American	4.73	1.01	.92	5.74	1.08	.91	3.54	1.36	.91	.405 (<.001)
Asian American	4.08	0.86	.92	4.70	0.90	.89	3.34	1.08	.88	.533 (<.001)
Native American	4.10	1.29	.96	4.80	1.36	.95	3.26	1.44	.94	.708 (<.001)
Latin American	4.01	1.06	.94	4.80	1.11	.93	3.08	1.32	.93	.536 (<.001)
Alcoholics	4.23	0.81	.86	4.88	0.87	.84	3.46	1.22	.88	.232 (<.001)
Poor People	4.60	0.93	.92	5.40	0.92	.89	3.65	1.30	.91	.467 (<.001)
Unemployed people	3.98	0.97	.92	4.76	0.96	.88	3.06	1.27	.91	.539 (<.001)
Overweight people	4.64	1.06	.94	5.34	0.97	.90	3.82	1.41	.93	.625 (<.001)
Wheelchair users	4.51	0.97	.92	5.13	0.94	.87	3.78	1.24	.90	.609 (<.001)
Transgender	5.04	0.88	.92	5.73	0.74	.87	4.21	1.35	.92	.444 (<.001)
Homosexual people	4.14	0.96	.92	4.90	0.91	.87	3.24	1.35	.92	.494 (<.001)
Conservatives	3.90	0.91	.91	4.69	0.90	.84	2.98	1.22	.91	.526 (<.001)
Liberals	3.82	0.86	.90	4.48	0.85	.84	3.05	1.11	.88	.587 (<.001)
Muslims	4.20	0.93	.91	4.96	0.92	.87	3.32	1.26	.89	.489 (<.001)
Jews	3.78	0.99	.93	4.44	1.01	.90	2.99	1.23	.92	.595 (<.001)
Atheists	3.41	1.01	.93	4.16	1.08	.89	2.53	1.20	.92	.591 (<.001)
Old people	3.93	0.74	.89	4.97	0.84	.87	2.69	1.03	.90	.277 (<.001)
Single parents	3.59	0.97	.93	4.35	1.06	.90	2.69	1.16	.91	.549 (<.001)

Note. The stigma consciousness scale includes the combined items of the stigma awareness and stigma anticipation subscale. The stigma awareness subscale consists of five items from the Stigma Consciousness Questionnaire (SCQ, Pinel, 1999) and eight items from the Stigmatization Scale (Harvey, 2000). The Stigma Anticipation subscale consists of four items of the SCQ and seven items from the Anticipated Stigma Scale (Quinn & Chaudoir, 2009).

Confirmatory factor analysis

Since we included items from different measures to develop a stigma consciousness scale with two latent factors, stigma awareness and stigma anticipation, we first conducted a confirmatory factor analysis testing this hypothesized factor structure. The two factors were allowed to correlate, and each item was constrained to load on its respective factor. Since assumptions regarding normality were violated on sample and subsample levels, we chose maximum likelihood with robust standard errors (MLR) as a robust estimation method for the CFA. Assumptions regarding multicollinearity and correlations of latent factors were met.

The hypothesized model demonstrated only poor (*RMSEA* = .111; *NFI* = .801; *TLI* = .784; *CFI* = .804) to marginal fit (*SRMR* = .080) to the data for both the sample and all subsamples (Table S.2). The only exception here were *SRMR* values for the Native American and Transgender subsample, where values between .050 and .080 suggested an acceptable fit.

Table S.2

Model fit for stigma consciousness two factor solution

**Group**	χ^2	***RMSEA***	***SRMR***	***NFI***	***TLI***	***CFI***
All	$\chi^2(251) = 12556.11$, $p < .001$	.111	.080	.801	.784	.804
African American	$\chi^2(251) = 975.112$, $p < .001$	.111	.104	.757	.787	.807
Asian American	$\chi^2(251) = 1051.40$, $p < .001$	.115	.121	.714	.741	.765
Native American	$\chi^2(251) = 590.43$, $p < .001$	.098	.061	.821	.876	.888
Latin American	$\chi^2(251) = 989.03$, $p < .001$	.112	.085	.798	.825	.841
Alcoholics	$\chi^2(251) = 1180.72$, $p < .001$	.124	.106	.665	.686	.714
Poor people	$\chi^2(251) = 658.78$, $p < .001$	.108	.081	.739	.771	.792
Unemployed people	$\chi^2(251) = 936.35$, $p < .001$	.109	.092	.754	.786	.806
Wheelchair users	$\chi^2(251) = 677.52$, $p < .001$	.105	.106	.715	.777	.797
Transgender	$\chi^2(251) = 890.41$, $p < .001$	.114	.075	.728	.766	.787

Homosexual people	$\chi^2(251) = 1013.73$, p <.001	.114	.091	.751	.779	.799
Conservatives	$\chi^2(251) = 997.09$, p <.001	.114	.095	.712	.742	.766
Liberals	$\chi^2(251) = 1239.83$, p <.001	.129	.104	.667	.685	.713
Muslims	$\chi^2(251) = 919.73$, p <.001	.109	.096	.734	.769	.790
Jews	$\chi^2(251) = 1090.52$, p <.001	.116	.087	.763	.786	.806
Atheists	$\chi^2(251) = 1103.06$, p <.001	.118	.083	.754	.777	.797
Old people	$\chi^2(251) = 1079.59$, p <.001	.118	.112	.703	.728	.753
Single parents	$\chi^2(251) = 857.32$, p <.001	.116	.101	.746	.784	.804
Overweight people	$\chi^2(251) = 903.90$, p <.001	.107	.081	.781	.814	.831

Table S.3 provides an overview of factor loadings and R^2 for the sample and each subsample. For the full sample, all items significantly loaded (p <.001) on their respective factor, but loadings varied greatly between items, ranging from .119 to .840 for stigma awareness and from .399 to .940 for stigma anticipation. The two factors stigma awareness and stigma anticipation significantly covaried at .516 (p <.001).

Table S.3

Confirmatory factor analysis of stigma consciousness two factor solution

**Group**	**Covariance**	**Item**	**Factor 1 (Stigma Awareness)**		**Factor 2 (Stigma Anticipation)**	
			Item loading	***R^2***	***Item loading***	***R^2***
All groups	.516 (<.001)	Item 1 (r)	.390 (<.001)	.152		
		Item 2	.526 (<.001)	.277		
		Item 3	.646 (<.001)	.418		
		Item 4	.715 (<.001)	.511		
		Item 5 (r)	.119 (<.001)	.014		
		Item 6	.766 (<.001)	.586		
		Item 7	.795 (<.001)	.632		

		Item 8	.829 (<.001	.687		
		Item 9	.840 (<.001	.706		
		Item 10	.754 (<.001)	.568		
		Item 11 (r)	.505 (<.001)	.255		
		Item 12 (r)	.479 (<.001)	.230		
		Item 13	.746 (<.001	.557		
		Item 14 (r)			.399 (<.001)	.159
		Item 15			.426 (<.001)	.181
		Item 16			.458 (<.001)	.210
		Item 17 (r)			.493 (<.001)	.243
		Item 18			.832 (<.001)	.693
		Item 19			.935 (<.001)	.874
		Item 20			.940 (<.001)	.883
		Item 21			.816 (<.001)	.666
		Item 22			.764 (<.001)	.583
		Item 23			.727 (<.001)	.528
		Item 24			.812 (<.001)	.659
African American	.348 (.003)	Item 1 (r)	.492 (<.001)	.242		
		Item 2	.666 (<.001)	.444		
		Item 3	.751 (<.001)	.564		
		Item 4	.805 (<.001)	.648		
		Item 5 (r)	.415 (<.001)	.172		
		Item 6	.747 (<.001)	.559		
		Item 7	.797 (<.001)	.636		
		Item 8	.857 (<.001)	.734		
		Item 9	.853 (<.001)	.728		
		Item 10	.767 (<.001)	.588		
		Item 11 (r)	.412 (<.001)	.170		
		Item 12 (r)	.301 (<.001)	.090		
		Item 13	.723 (<.001)	.523		

		Item 14 (r)			.337 (<.001)	.114
		Item 15			.375 (<.001)	.141
		Item 16			.368 (<.001)	.135
		Item 17 (r)			.436 (<.001)	.191
		Item 18			.823 (<.001)	.677
		Item 19			.912 (<.001)	.831
		Item 20			.910 (<.001)	.827
		Item 21			.863 (<.001)	.745
		Item 22			.768 (<.001)	.590
		Item 23			.747 (<.001)	.558
		Item 24			.830 (<.001)	.689
Asian American	.416 (.003)	Item 1 (r)	.595 (<.001)	.353		
		Item 2	.459 (<.001)	.211		
		Item 3	.713 (<.001)	.509		
		Item 4	.806 (<.001)	.649		
		Item 5 (r)	.195 (<.001)	.038		
		Item 6	.684 (<.001)	.468		
		Item 7	.751 (<.001)	.564		
		Item 8	.790 (<.001)	.624		
		Item 9	.874 (<.001)	.767		
		Item 10	.713 (<.001)	.510		
		Item 11 (r)	.538 (<.001)	.290		
		Item 12 (r)	.465 (<.001)	.216		
		Item 13	.659 (<.001)	.434		
		Item 14 (r)			.374 (<.001)	.140
		Item 15			.307 (<.001)	.094
		Item 16			.309 (<.001)	.096
		Item 17 (r)			.379 (<.001)	.144
		Item 18			.756 (<.001)	.572
		Item 19			.942 (<.001)	.887

		Item 20			.930 (<.001)	.865
		Item 21			.768 (<.001)	.590
		Item 22			.656 (<.001)	.430
		Item 23			.649 (<.001)	.421
		Item 24			.699 (<.001)	.489
Native American	.705 (<.001)	Item 1 (r)	.874 (<.001)	.763		
		Item 2	.691 (<.001)	.478		
		Item 3	.710 (<.001)	.504		
		Item 4	.836 (<.001)	.699		
		Item 5 (r)	.427 (<.001)	.182		
		Item 6	.774 (<.001)	.600		
		Item 7	.877 (<.001)	.768		
		Item 8	.893 (<.001)	.798		
		Item 9	.880 (<.001)	.775		
		Item 10	.857 (<.001)	.734		
		Item 11 (r)	.688 (<.001)	.473		
		Item 12 (r)	.708 (<.001)	.501		
		Item 13	.787 (<.001)	.620		
		Item 14 (r)			.451 (<.001)	.204
		Item 15			.694 (<.001)	.482
		Item 16			.500 (<.001)	.250
		Item 17 (r)			.696 (<.001)	.484
		Item 18			.857 (<.001)	.734
		Item 19			.948 (<.001)	.898
		Item 20			.968 (<.001)	.937
		Item 21			.882 (<.001)	.778
		Item 22			.765 (<.001)	.585
		Item 23			.759 (<.001)	.576
		Item 24			.770	.592
	.510 (<.001)	Item 1 (r)	.761 (<.001)	.579		

Latin American		Item 2	.452 (<.001)	.205		
		Item 3	.807 (<.001)	.652		
		Item 4	.868 (<.001)	.754		
		Item 5 (r)	.436 (<.001)	.190		
		Item 6	.774 (<.001)	.598		
		Item 7	.851 (<.001)	.724		
		Item 8	.770 (<.001)	.594		
		Item 9	.852 (<.001)	.726		
		Item 10	.773 (<.001)	.598		
		Item 11 (r)	.602 (<.001)	.362		
		Item 12 (r)	.644 (<.001)	.415		
		Item 13	.812 (<.001)	.659		
		Item 14 (r)			.446 (<.001)	.199
		Item 15			.541 (<.001)	.293
		Item 16			.545 (<.001)	.297
		Item 17 (r)			.477 (<.001)	.227
		Item 18			.885 (<.001)	.784
		Item 19			.958 (<.001)	.918
		Item 20			.937 (<.001)	.877
		Item 21			.894 (<.001)	.799
		Item 22			.776 (<.001)	.602
		Item 23			.758 (<.001)	.575
		Item 24			.799 (<.001)	.638
Alcoholics	.141 (.401)	Item 1 (r)	.385 (<.001)	0.148		
		Item 2	.503 (<.001)	0.253		
		Item 3	.673 (<.001)	0.453		
		Item 4	.753 (<.001)	0.567		
		Item 5 (r)	-.013 (.882)	0.000		
		Item 6	.676 (<.001)	0.457		

		Item 7	.671 (<.001)	0.450		
		Item 8	.708 (<.001)	0.502		
		Item 9	.774 (<.001)	0.598		
		Item 10	.734 (<.001)	0.539		
		Item 11 (r)	.253 (<.001)	0.064		
		Item 12 (r)	.227 (.001)	0.051		
		Item 13	.681 (.006)	0.464		
		Item 14 (r)			.104 (.104)	0.011
		Item 15			.239 (.104)	0.057
		Item 16			.455 (.220)	0.207
		Item 17 (r)			.299 (.125)	0.089
		Item 18			.769 (.213)	0.591
		Item 19			.904 (.209)	0.817
		Item 20			.890 (.207)	0.792
		Item 21			.796 (.221)	0.633
		Item 22			.817 (.230)	0.667
		Item 23			.754 (.238)	0.568
		Item 24			.848 (.215)	0.720
Poor people	.455 (<.001)	Item 1 (r)	.560 (<.001)	0.314		
		Item 2	.391 (<.001)	0.153		
		Item 3	.681 (<.001)	0.464		
		Item 4	.679 (<.001)	0.461		
		Item 5 (r)	.336 (<.001)	0.113		
		Item 6	.695 (<.001)	0.483		
		Item 7	.744 (<.001)	0.554		
		Item 8	.825 (<.001)	0.681		
		Item 9	.801 (<.001)	0.641		
		Item 10	.739 (<.001)	0.546		
		Item 11 (r)	.652 (<.001)	0.426		
		Item 12 (r)	.349 (<.001)	0.122		

		Item 13	.788 (<.001)	0.621		
		Item 14 (r)			.423 (<.001)	0.179
		Item 15			.389 (<.001)	0.152
		Item 16			.442 (<.001)	0.196
		Item 17 (r)			.539 (<.001)	0.290
		Item 18			.762 (<.001)	0.580
		Item 19			.918 (<.001)	0.842
		Item 20			.954 (<.001)	0.910
		Item 21			.760 (<.001)	0.577
		Item 22			.680 (<.001)	0.462
		Item 23			.644 (<.001)	0.415
		Item 24			.746 (<.001)	0.557
Unemployed people	.538 (.002)	Item 1 (r)	.411 (<.001)	0.169		
		Item 2	.584 (<.001)	0.341		
		Item 3	.759 (<.001)	0.577		
		Item 4	.776 (<.001)	0.602		
		Item 5 (r)	-.021 (.805)	0.000		
		Item 6	.773 (<.001)	0.598		
		Item 7	.785 (<.001)	0.616		
		Item 8	.804 (<.001)	0.646		
		Item 9	.820 (<.001)	0.673		
		Item 10	.663 (<.001)	0.440		
		Item 11 (r)	.501 (<.001)	0.251		
		Item 12 (r)	.276 (.001)	0.076		
		Item 13	.772 (<.001)	0.596		
		Item 14 (r)			.386 (<.001)	0.149
		Item 15			.447 (<.001)	0.200
		Item 16			.605 (<.001)	0.366
		Item 17 (r)			.546 (<.001)	0.298
		Item 18			.891 (<.001)	0.795

		Item 19			.950 (<.001)	0.903
		Item 20			.939 (<.001)	0.882
		Item 21			.751 (<.001)	0.564
		Item 22			.673 (<.001)	0.453
		Item 23			.582 (<.001)	0.338
		Item 24			.762 (<.001)	0.581
Wheelchair users	.536 (.015)	Item 1 (r)	.540 (<.001)	0.291		
		Item 2	.314 (.002)	0.099		
		Item 3	.665 (<.001)	0.442		
		Item 4	.839 (<.001)	0.704		
		Item 5 (r)	.313 (.001)	0.098		
		Item 6	.742 (<.001)	0.551		
		Item 7	.790 (<.001)	0.624		
		Item 8	.744 (<.001)	0.553		
		Item 9	.770 (<.001)	0.594		
		Item 10	.693 (<.001)	0.480		
		Item 11 (r)	.510 (<.001)	0.260		
		Item 12 (r)	.550 (<.001)	0.303		
		Item 13	.534 (<.001)	0.285		
		Item 14 (r)			.321 (<.001)	0.103
		Item 15			.452 (<.001)	0.204
		Item 16			.437 (.003)	0.191
		Item 17 (r)			.348 (<.001)	0.121
		Item 18			.825 (.001)	0.681
		Item 19			.906 (<.001)	0.822
		Item 20			.938 (<.001)	0.881
		Item 21			.873 (<.001)	0.763
		Item 22			.650 (.001)	0.422
		Item 23			.589 (.001)	0.348
		Item 24			.739 (<.001)	0.547

Transgender	.455 (.004)	Item 1 (r)	.725 (<.001)	0.525		
		Item 2	.326 (<.001)	0.106		
		Item 3	.510 (<.001)	0.260		
		Item 4	.743 (<.001)	0.552		
		Item 5 (r)	.434 (<.001)	0.188		
		Item 6	.612 (<.001)	0.375		
		Item 7	.650 (<.001)	0.423		
		Item 8	.700 (<.001)	0.490		
		Item 9	.715 (<.001)	0.511		
		Item 10	.565 (<.001)	0.320		
		Item 11 (r)	.630 (<.001)	0.397		
		Item 12 (r)	.566 (<.001)	0.320		
		Item 13	.558 (<.001)	0.312		
		Item 14 (r)			.274 (<.001)	0.075
		Item 15			.526 (<.001)	0.277
		Item 16			.334 (.002)	0.112
		Item 17 (r)			.540 (<.001)	0.291
		Item 18			.831 (.001)	0.691
		Item 19			.953 (.001)	0.909
		Item 20			.957 (.001)	0.916
		Item 21			.815 (.001)	0.663
		Item 22			.841 (<.001)	0.707
		Item 23			.802 (<.001)	0.643
		Item 24			.849 (<.001)	0.720
Homosexual people	.474 (<.001)	Item 1 (r)	0.547 (<.001)	0.300		
		Item 2	0.289 (<.001)	0.083		
		Item 3	0.600 (<.001)	0.360		
		Item 4	0.754 (<.001)	0.568		
		Item 5 (r)	0.154 (.051)	0.024		
		Item 6	0.768 (<.001)	0.590		

		Item 7	0.740 (<.001)	0.548		
		Item 8	0.820 (<.001)	0.672		
		Item 9	0.803 (<.001)	0.645		
		Item 10	0.552 (<.001)	0.304		
		Item 11 (r)	0.437 (<.001)	0.191		
		Item 12 (r)	0.323 (<.001)	0.104		
		Item 13	0.784 (<.001)	0.615		
		Item 14 (r)			0.363 (<.001)	0.132
		Item 15			0.442 (<.001)	0.195
		Item 16			0.493 (<.001)	0.243
		Item 17 (r)			0.447 (<.001)	0.200
		Item 18			0.871 (<.001)	0.758
		Item 19			0.920 (<.001)	0.847
		Item 20			0.950 (<.001)	0.903
		Item 21			0.891 (<.001)	0.794
		Item 22			0.819 (<.001)	0.670
		Item 23			0.832 (<.001)	0.692
		Item 24			0.838 (<.001)	0.703
Conservatives	.562 (.026)	Item 1 (r)	.259 (.002)	0.067		
		Item 2	.501 (.002)	0.251		
		Item 3	.497 (<.001)	0.247		
		Item 4	.570 (<.001)	0.325		
		Item 5 (r)	.045 (.587)	0.002		
		Item 6	.717 (.003)	0.514		
		Item 7	.769 (.001)	0.591		
		Item 8	.760 (.002)	0.578		
		Item 9	.713 (.002)	0.508		
		Item 10	.696 (.005)	0.485		
		Item 11 (r)	.401 (.002)	0.161		
		Item 12 (r)	.374 (.006)	0.140		

		Item 13	.660 (.003)	0.436		
		Item 14 (r)			.296 (<.001)	0.088
		Item 15			.370 (<.001)	0.137
		Item 16			.472 (.001)	0.223
		Item 17 (r)			.366 (<.001)	0.134
		Item 18			.784 (<.001)	0.614
		Item 19			.885 (<.001)	0.783
		Item 20			.861 (<.001)	0.741
		Item 21			.832 (<.001)	0.692
		Item 22			.880 (.001)	0.774
		Item 23			.855 (.001)	0.731
		Item 24			.874 (<.001)	0.764
Liberals	.619 (.644)	Item 1 (r)	.045 (.630)	.002		
		Item 2	.342 (.632)	.117		
		Item 3	.267 (.614)	.071		
		Item 4	.410 (.617)	.168		
		Item 5 (r)	-.106 (.697)	.011		
		Item 6	.800 (.635)	.639		
		Item 7	.827 (.635)	.684		
		Item 8	.721 (.632)	.520		
		Item 9	.630 (.629)	.397		
		Item 10	.774 (.633)	.600		
		Item 11 (r)	.529 (.617)	.280		
		Item 12 (r)	.565 (.617)	.319		
		Item 13	.565 (.625)	.601		
		Item 14 (r)			.272 (<.001)	.074
		Item 15			.078 (.284)	.006
		Item 16			.410 (.002)	.168
		Item 17 (r)			.263 (<.001)	.069
		Item 18			.809 (<.001)	.654

		Item 19			.927 (.001)	.878
		Item 20			.938 (<.001)	.880
		Item 21			.722 (.001)	.521
		Item 22			.788 (<.001)	.621
		Item 23			.771 (<.001)	.594
		Item 24			.806 (<.001)	.650
Muslims	.412 (.058)	Item 1 (r)	.413 (<.001)	0.170		
		Item 2	.260 (.009)	0.067		
		Item 3	.673 (<.001)	0.453		
		Item 4	.610 (<.001)	0.372		
		Item 5 (r)	.263 (<.001)	0.069		
		Item 6	.657 (<.001)	0.432		
		Item 7	.766 (<.001)	0.587		
		Item 8	.775 (<.001)	0.600		
		Item 9	.839 (<.001)	0.704		
		Item 10	.692 (<.001)	0.479		
		Item 11 (r)	.405 (<.001)	0.164		
		Item 12 (r)	.452 (<.001)	0.204		
		Item 13	.743 (<.001)	0.552		
		Item 14 (r)			.210 (<.001)	0.044
		Item 15			.298 (.001)	0.089
		Item 16			.389 (.013)	0.151
		Item 17 (r)			.364 (.001)	0.133
		Item 18			.764 (.010)	0.584
		Item 19			.904 (.009)	0.818
		Item 20			.904 (.009)	0.818
		Item 21			.829 (.006)	0.687
		Item 22			.874 (.007)	0.764
		Item 23			.848 (.007)	0.720
		Item 24			.823 (.007)	0.677

Jews	.577 (<.001)	Item 1 (r)	.586 (<.001)	0.344		
		Item 2	.334 (<.001)	0.111		
		Item 3	.605 (<.001)	0.367		
		Item 4	.810 (<.001)	0.656		
		Item 5 (r)	.159 (.024)	0.025		
		Item 6	.772 (<.001)	0.596		
		Item 7	.636 (<.001)	0.404		
		Item 8	.798 (<.001)	0.638		
		Item 9	.879 (<.001)	0.772		
		Item 10	.673 (<.001)	0.453		
		Item 11 (r)	.731 (<.001)	0.534		
		Item 12 (r)	.384 (<.001)	0.148		
		Item 13	.791 (<.001)	0.626		
		Item 14 (r)			.337 (<.001)	0.114
		Item 15			.353 (<.001)	0.125
		Item 16			.529 (<.001)	0.280
		Item 17 (r)			.400 (<.001)	0.160
		Item 18			.866 (<.001)	0.750
		Item 19			.973 (<.001)	0.946
		Item 20			.980 (<.001)	0.960
		Item 21			.780 (<.001)	0.609
		Item 22			.754 (<.001)	0.569
		Item 23			.731 (<.001)	0.535
		Item 24			.829 (<.001)	0.687
Atheists	.617 (<.001)	Item 1 (r)	.445 (<.001)	0.198		
		Item 2	.472 (<.001)	0.223		
		Item 3	.489 (<.001)	0.239		
		Item 4	.675 (<.001)	0.455		
		Item 5 (r)	-.048 (.547)	0.002		
		Item 6	.817 (<.001)	0.668		

		Item 7	.853 (<.001)	0.728		
		Item 8	.783 (<.001)	0.614		
		Item 9	.818 (<.001)	0.669		
		Item 10	.740 (<.001)	0.548		
		Item 11 (r)	.620 (<.001)	0.385		
		Item 12 (r)	.568 (<.001)	0.323		
		Item 13	.804 (<.001)	0.646		
		Item 14 (r)			.439 (<.001)	0.193
		Item 15			.426 (<.001)	0.181
		Item 16			.557 (<.001)	0.310
		Item 17 (r)			.625 (<.001)	0.390
		Item 18			.875 (<.001)	0.766
		Item 19			.974 (<.001)	0.950
		Item 20			.970 (<.001)	0.941
		Item 21			.707 (<.001)	0.500
		Item 22			.674 (<.001)	0.455
		Item 23			.673 (<.001)	0.453
		Item 24			.786 (<.001)	0.617
Old people	.220 (.031)	Item 1 (r)	.506 (<.001)	0.256		
		Item 2	.479 (<.001)	0.229		
		Item 3	.727 (<.001)	0.528		
		Item 4	.778 (<.001)	0.605		
		Item 5 (r)	.316 (<.001)	0.100		
		Item 6	.689 (<.001)	0.474		
		Item 7	.692 (<.001)	0.478		
		Item 8	.681 (<.001)	0.464		
		Item 9	.775 (<.001)	0.600		
		Item 10	.621 (<.001)	0.386		
		Item 11 (r)	.429 (<.001)	0.184		
		Item 12 (r)	.412 (<.001)	0.170		

		Item 13	.592 (<.001)	0.350		
		Item 14 (r)			.286 (<.001)	0.082
		Item 15			.330 (<.001)	0.109
		Item 16			.509 (<.001)	0.259
		Item 17 (r)			.318 (<.001)	0.101
		Item 18			.772 (<.001)	0.596
		Item 19			.913 (<.001)	0.833
		Item 20			.945 (<.001)	0.893
		Item 21			.907 (<.001)	0.822
		Item 22			.781 (<.001)	0.611
		Item 23			.731 (<.001)	0.535
		Item 24			.744 (<.001)	0.553
Single parents	.516 (<.001)	Item 1 (r)	.558 (<.001)	0.311		
		Item 2	.545 (<.001)	0.297		
		Item 3	.674 (<.001)	0.454		
		Item 4	.823 (<.001)	0.677		
		Item 5 (r)	-.302 (.006)	0.091		
		Item 6	.801 (<.001)	0.642		
		Item 7	.826 (<.001)	0.682		
		Item 8	.858 (<.001)	0.736		
		Item 9	.842 (<.001)	0.709		
		Item 10	.807 (<.001)	0.651		
		Item 11 (r)	.530 (<.001)	0.281		
		Item 12 (r)	.469 (<.001)	0.220		
		Item 13	.827 (<.001)	0.684		
		Item 14 (r)			.538 (<.001)	0.290
		Item 15			.484 (<.001)	0.234
		Item 16			.498 (<.001)	0.248
		Item 17 (r)			.605 (<.001)	0.366
		Item 18			.872 (<.001)	0.761

		Item 19			.966 (<.001)	0.934
		Item 20			.929 (<.001)	0.864
		Item 21			.743 (<.001)	0.553
		Item 22			.623 (<.001)	0.389
		Item 23			.577 (<.001)	0.333
		Item 24			.748 (<.001)	0.559
Overweight people	.603 (<.001)	Item 1 (r)	.466 (<.001)	0.218		
		Item 2	.676 (<.001)	0.457		
		Item 3	.748 (<.001)	0.560		
		Item 4	.820 (<.001)	0.673		
		Item 5 (r)	.134 (.089)	0.018		
		Item 6	.747 (<.001)	0.558		
		Item 7	.726 (<.001)	0.528		
		Item 8	.819 (<.001)	0.671		
		Item 9	.801 (<.001)	0.642		
		Item 10	.802 (<.001)	0.642		
		Item 11 (r)	.547 (<.001)	0.299		
		Item 12 (r)	.509 (<.001)	0.260		
		Item 13	.811 (<.001)	0.658		
		Item 14 (r)			.519 (<.001)	0.269
		Item 15			.489 (<.001)	0.239
		Item 16			.662 (<.001)	0.439
		Item 17 (r)			.590 (<.001)	0.348
		Item 18			.833 (<.001)	0.693
		Item 19			.937 (<.001)	0.878
		Item 20			.934 (<.001)	0.873
		Item 21			.820 (<.001)	0.672
		Item 22			.720 (<.001)	0.518
		Item 23			.692 (<.001)	0.478
		Item 24			.809 (<.001)	0.655

Note. Items 1-5 and 14-17 from the Stigma Consciousness Questionnaire (SCQ, Pinel, 1999), items 6-13 from the Stigmatization Scale (Harvey, 2000), items 18-24 from the Anticipated Stigma Scale (Quinn & Chaudoir, 2009). A list of all items with item number and wording is reported in Appendix S1, Table S.5.

Post-hoc modifications and adaptions

Given the insufficient model fit of the preregistered factor solution and low factor loadings (<.50) of several items, we conducted post hoc modifications to the preregistered two-factor model in an effort to identify a better fitting factor solution. Note that these adaptions were entirely data driven, based on modifications indices, investigation of item loadings and exploratory factor analyses for underlying factor structure.

First, we explored possible adaptions to the preregistered model by examining modifications indices, item loadings and performing item analyses. Modification indices at sample and at subsample level indicated high error covariances of two items of the anticipation scale (item nine and ten) across all subsamples. We ran confirmatory factor analyses for two models, each excluding one of these items, to investigate the model fit (Models A and B in Table S.4). Since the adapted models are based on a subset of the items of the preregistered model, we cannot statistically compare the model fit. However, results indicate that the adapted models do not provide good fit for the overall sample, with fit indices ranging from poor to marginal for each subsample. Further improvements per subsample based on modification indices were group specific, in that no adaptions could be equally applied to all subsamples. Since we want to compare stigma consciousness levels between subsamples, we decided to not include group specific modifications.

We then investigated the results of the factor analysis on item level for items that either correlated highly with another item (r >.80, p <.050) or showed low factor loadings (<.50), indicating that these items should be excluded from the model. Again, there were no systematics across subsamples, so we decided to not exclude items to ensure we measure the same construct for all groups.

We further explored item correlations, item means and variances, item difficulty and Cronbach's α. For the overall sample as well as within each subsample, item six and seven as well as item nine and ten of the anticipation subscale correlated highly (r > .80, p <.050). We ran confirmatory factor analyses for four alternative models, each excluding one of these items (Model C and Model D in Table S.4). Since the

adapted models are based on a subset of items used for the preregistered model, we cannot statistically compare the models regarding their model fit. However, as presented in Table S.4, the model fit does not seem to improve for any of the adapted models.

We additionally ran exploratory factor analyses across the sample and per subsample to investigate the underlying factor structure. We employed parallel analyses for factor number estimation. Analyses revealed a seven-factor solution for the sample, and two, three, four or five factor solutions for the subsamples. We ran confirmatory factor analyses for the models suggested by EFA across all groups and per subsample and compared the model fit with the preregistered two factor solution. The models based on EFA showed significantly better fit to the data compared to the preregistered two factor model for the overall sample as well as each subsample except for Native Americans, as indicated by the results of the χ^2 difference tests (Table S.4). However, we did not find a factor solution that could be applied equally to all subsamples, thus not allowing group comparisons regarding stigma consciousness in further analyses.

Table S.4

Model fit of adapted models of stigma consciousness measure per subsample

Group	*Model*	χ^2	*RMSEA*	*SRMR*	*NFI*	*TLI*	*CFI*	*BIC*	*AIC*	χ^2 *difference of Model SC and EFA Model*
All groups	Model SC	$\chi^2(251) = 12556.11$, $p < .001$	.111	.080	.801	.784	.804	319007.65	318548.74	$\chi^2(19, 251) = 5427.10$, $p < .001$
	EFA: Seven Factor	$\chi^2(232) = 5419.28$, $p < .001$	.075	.047	.914	.902	.917	312028.26	311449.93	
	A	$\chi^2(229) = 9549.66$, $p < .001$	.101	.082	.832	.818	.835	306780.99	306340.95	
	B	$\chi^2(229) = 9437.61$, $p < .001$	.101	.081	.835	.821	.838	306539.08	306099.05	
	C	$\chi^2(229) = 11169.33$, $p < .001$	.110	.080	.797	.780	.801	309426.38	308986.34	
	D	$\chi^2(229) = 10783.84$, $p < .001$	.108	.081	.804	.787	.807	309161.36	308721.32	
African American	Model SC	$\chi^2(251) = 975.11$, $p < .001$	.111	.104	.757	.787	.807	19120.51	18868.58	$\chi^2(2, 251) = 274.35$, $p < .001$
	EFA: Three Factor	$\chi^2(249) = 726.73$, $p < .001$	.091	.072	.819	.859	.872	18883.03	18624.20	
	A	$\chi^2(229) = 772.96$, $p < .001$	.101	.105	.787	.822	.839	18361.19	18119.61	

	B	$\chi^2(229) = 751.74$, p <.001	.099	.105	.793	.829	.845	18343.31	18101.74	
	C	$\chi^2(229) = 903.44$, p <.001	.112	.106	.750	.778	.799	18470.38	18228.80	
	D	$\chi^2(229) = 872.01$, p <.001	.110	.106	.757	.787	.807	18474.62	18233.05	
Asian American	Model SC	$\chi^2(251) = 1051.40$, p <.001	.115	.121	.714	.741	.765	18498.25	18243.86	$\chi^2(4, 251) = 247.16$, p <.001
	EFA: Four factor	$\chi^2(247) = 786.98$, p <.001	.095	.078	.786	.823	.841	18255.78	17987.45	
	A	$\chi^2(229) = 887.80$, p <.001	.109	.124	.738	.768	.790	17727.05	17483.12	
	B	$\chi^2(229) = 862.95$, p <.001	.107	.123	.744	.775	.797	17744.84	17500.90	
	C	$\chi^2(229) = 949.26$, p <.001	.114	.112	.706	.732	.758	17919.41	17675.48	
	D	$\chi^2(229) = 918.73$, p <.001	.112	.112	.712	.740	.765	17938.69	17694.76	
Native American	Model SC	$\chi^2(251) = 590.43$, p <.001	.098	.061	.821	.876	.888	10998.74	10784.00	*Models have the same df, therefore a χ^2-difference test is not possible. For model comparison see AIC values.*
	EFA: Two Factor	$\chi^2(251) = 583.54$, p <.001	.097	.058	.823	.879	.890	10991.85	10777.11	
	A	$\chi^2(229) = 465.14$, p <.001	.086	.062	.848	.907	.916	10552.26	10346.35	
	B	$\chi^2(229) = 474.61$, p <.001	.088	.062	.845	.904	.913	10559.72	10353.80	
	C	$\chi^2(229) = 425.03$, p <.001	.091	.056	.830	.889	.900	10711.18	10505.26	
	D	$\chi^2(229) = 477.36$, p <.001	.088	.056	.837	.897	.907	10675.34	10469.43	
Latin American	Model SC	$\chi^2(251) = 989.03$, p <.001	.112	.085	.798	.825	.841	17686.71	17433.85	$\chi^2(2, 251) = 151.84$, p <.001
	EFA: Three Factor	$\chi^2(249) = 798.64$, p <.001	.097	.069	.837	.868	.881	17507.25	17247.46	
	A	$\chi^2(229) = 819.57$, p <.001	.105	.085	.819	.848	.862	16992.25	16749.78	
	B	$\chi^2(229) = 804.73$, p <.001	.103	.085	.822	.851	.865	16996.42	16753.95	
	C	$\chi^2(229) = 889.77$, p <.001	.111	.085	.797	.823	.840	17148.57	16906.10	
	D	$\chi^2(253) = 872.17$, p <.001	.109	.086	.798	.826	.842	17211.68	16969.21	
Alcoholic s	Model SC	$\chi^2(251) = 1180.72$, p <.001	.124	.106	.665	.686	.714	19956.19	19702.11	$\chi^2(5, 251) = 230.03$, p <.001
	EFA: Four Factor	$\chi^2(246) = 880.53$, p <.001	.104	.079	.750	.781	.805	19683.41	19412.00	
	A	$\chi^2(229) = 946.82$, p <.001	.114	.106	.693	.719	.746	19202.39	18958.75	
	B	$\chi^2(229) = 965.89$, p <.001	.116	.106	.693	.719	.745	19181.50	18937.85	
	C	$\chi^2(229) = 1014.02$, p <.001	.120	.111	.672	.694	.723	19255.37	19011.73	
	D	$\chi^2(229) = 1015.32$, p <.001	.120	.112	.670	.692	.721	19246.33	19002.68	
Poor people	Model SC	$\chi^2(251) = 658.78$, p <.001	.108	.081	.739	.771	.792	19049.74	18795.65	$\chi^2(5, 251) = 263.17$, p <.001
	EFA: Four factor	$\chi^2(246) = 802.96$, p <.001	.097	.075	.782	.816	.836	18921.32	18649.84	

	A	$\chi^2(229) = 687.27$, p <.001	.091	.078	.790	.832	.848	18233.17	17989.52	
	B	$\chi^2(229) = 687.74$, p <.001	.091	.079	.791	.833	.849	18241.61	17997.97	
	C	$\chi^2(229) = 808.04$, p <.001	.103	.079	.744	.779	.800	18416.21	18172.57	
	D	$\chi^2(229) = 804.85$, p <.001	.102	.079	.749	.785	.805	18358.81	18115.16	
Unemployed people	Model SC	$\chi^2(251) = 936.35$, p <.001	.109	.092	.754	.786	.806	18144.73	17894.39	$\chi^2(2, 251) = 94.91$, p <.001
	EFA: Three factor	$\chi^2(249) = 770.48$, p <.001	.096	.075	.797	.836	.852	17989.72	17732.52	
	A	$\chi^2(229) = 719.75$, p <.001	.097	.089	.792	.831	.847	17447.95	17207.90	
	B	$\chi^2(229) = 669.21$, p <.001	.092	.085	.806	.848	.862	17454.84	17214.79	
	C	$\chi^2(229) = 888.42$, p <.001	.112	.094	.735	.765	.787	17594.19	17354.13	
	D	$\chi^2(229) = 836.72$, p <.001	.108	.096	.746	.779	.800	17583.97	17343.91	
Wheelchair users	Model SC	$\chi^2(251) = 677.52$, p <.001	.105	.106	.715	.777	.797	12479.92	12258.22	$\chi^2(2, 251) = 149.90$, p <.001
	EFA: Three factor	$\chi^2(249) = 516.08$, p <.001	.083	.064	.783	.859	.873	12328.56	12100.78	
	A	$\chi^2(229) = 533.04$, p <.001	.093	.106	.753	.824	.840	11953.32	11740.73	
	B	$\chi^2(229) = 538.27$, p <.001	.094	.106	.753	.823	.839	11932.38	11719.79	
	C	$\chi^2(229) = 616.51$, p <.001	.105	.102	.701	.763	.786	12090.35	11877.77	
	D	$\chi^2(229) = 619.20$, p <.001	.105	.106	.705	.766	.788	12057.40	11844.81	
Transgender	Model SC	$\chi^2(251) = 890.41$, p <.001	.114	.075	.728	.766	.787	14570.54	14331.24	$\chi^2(5, 251) = 72.10$, p <.001
	EFA: Four factor	$\chi^2(246) = 722.66$, p <.001	.099	.065	.780	.822	.841	14429.19	14173.50	
	A	$\chi^2(229) = 723.43$, p <.001	.105	.076	.750	.793	.813	13979.62	13750.15	
	B	$\chi^2(229) = 703.63$, p <.001	.103	.076	.758	.802	.821	13942.15	13712.68	
	C	$\chi^2(229) = 742.36$, p <.001	.107	.075	.733	.776	.797	14100.21	13870.74	
	D	$\chi^2(229) = 726.10$, p <.001	.105	.075	.739	.783	.803	14091.61	13862.14	
Homosexual people	Model SC	$\chi^2(251) = 1013.73$, p <.001	.114	.091	.751	.779	.799	18383.83	18131.90	$\chi^2(5,251) = 203.32$, p <.001
	EFA: Four Factor	$\chi^2(246) = 712.54$, p <.001	.090	.064	.825	.862	.877	18109.89	17840.71	
	A	$\chi^2(229) = 867.84$, p <.001	.109	.093	.764	.794	.814	17682.02	17440.45	
	B	$\chi^2(229) = 851.86$, p <.001	.108	.093	.767	.797	.817	17697.10	17455.52	
	C	$\chi^2(229) = 949.97$, p <.001	.116	.092	.733	.759	.782	17879.08	17637.51	
	D	$\chi^2(229) = 898.33$, p <.001	.112	.092	.749	.777	.798	17787.10	17545.53	
	Model SC	$\chi^2(251) = 997.09$, p <.001	.114	.095	.712	.742	.766	18780.46	18529.48	$\chi^2(9, 251) = 166.09$,

Conservatives	EFA: Five Factor	$\chi^2(242) = 758.87, p < .001$	.096	.088	.781	.815	.838	18591.21	18309.29	$p < .001$
	A	$\chi^2(229) = 837.22, p < .001$	.107	.096	.723	.757	.780	18162.31	17921.64	
	B	$\chi^2(229) = 813.76, p < .001$	.105	.095	.731	.767	.789	18121.53	17880.87	
	C	$\chi^2(229) = 863.89, p < .001$	.110	.098	.718	.751	.774	18129.47	17888.80	
	D	$\chi^2(229) = 856.72, p < .001$	.109	.099	.717	.750	.774	18144.79	17904.12	
Liberals	Model SC	$\chi^2(251) = 1239.83, p < .001$	.129	.104	.667	.685	.713	18868.98	18615.81	$\chi^2(9, 251) = 178.90$ $p < .001$
	EFA: Five Factor	$\chi^2(242) = 889.42, p < .001$	.106	.085	.761	.786	.812	18567.78	18283.40	
	A	$\chi^2(229) = 1082.06, p < .001$	.125	.108	.678	.697	.726	18174.11	17931.35	
	B	$\chi^2(229) = 1050.00, p < .001$	.123	.107	.686	.707	.735	18179.73	17936.97	
	C	$\chi^2(229) = 1028.73, p < .001$	.121	.109	.678	.700	.728	18253.90	18011.13	
	D	$\chi^2(229) = 995.71, p < .001$	.119	.108	.686	.710	.737	18238.14	17995.38	
Muslims	Model SC	$\chi^2(251) = 919.73, p < .001$	.109	.096	.734	.769	.790	18442.43	18192.73	$\chi^2(2, 251) = 193.26,$ $p < .001$
	EFA: Three factor	$\chi^2(249) = 695.14, p < .001$	.089	.062	.799	.844	.860	18228.68	17972.14	
	A	$\chi^2(229) = 817.82, p < .001$	.107	.102	.735	.770	.792	17813.45	17574.01	
	B	$\chi^2(229) = 800.46, p < .001$	.105	.101	.742	.778	.799	17769.75	17530.32	
	C	$\chi^2(229) = 763.49, p < .001$	.102	.096	.745	.785	.805	17820.81	17581.37	
	D	$\chi^2(229) = 739.11, p < .001$	.099	.096	.752	.793	.813	17830.40	17590.97	
Jews	Model SC	$\chi^2(251) = 1090.52, p < .001$	.116	.087	.763	.786	.806	19024.81	18768.63	$\chi^2(4, 251) = 127.89,$ $p < .001$
	EFA: Four Factor	$\chi^2(247) = 921.70, p < .001$	.105	.066	.799	.825	.844	18878.03	18607.81	
	A	$\chi^2(229) = 764.96, p < .001$	.097	.087	.812	.845	.860	18256.86	18011.20	
	B	$\chi^2(229) = 756.02, p < .001$	.097	.087	.815	.848	.862	18231.53	17985.87	
	C	$\chi^2(229) = 923.46, p < .001$	.111	.083	.763	.789	.809	18596.24	18350.59	
	D	$\chi^2(229) = 902.98, p < .001$	.109	.083	.768	.796	.815	18580.31	18334.65	
Atheists	Model SC	$\chi^2(251) = 1103.06, p < .001$	.118	.083	.754	.777	.797	19110.41	18855.12	$\chi^2(9, 251) = 198.34,$ $p < .001$
	EFA: Five factor	$\chi^2(242) = 713.66, p < .001$	.089	.068	.841	.872	.888	18770.48	18483.71	
	A	$\chi^2(229) = 920.23, p < .001$	.111	.081	.779	.804	.823	18394.83	18150.03	
	B	$\chi^2(229) = 917.51, p < .001$	.111	.081	.779	.805	.824	18432.76	18187.96	
	C	$\chi^2(229) = 940.33, p < .001$	.113	.080	.755	.780	.801	18639.52	18394.71	
	D	$\chi^2(229) = 940.35, p < .001$	.113	.080	.754	.780	.801	18650.23	18405.43	

Old people	Model SC	$\chi^2(251) = 1079.59, p <.001$	.118	.112	.703	.728	.753	17636.55	17383.38	$\chi^2(2, 251) = 89.91, p <.001$
	EFA: Three factor	$\chi^2(249) = 872.39, p <.001$	.103	.098	.760	.794	.814	17440.27	17180.17	
	A	$\chi^2(229) = 883.57, p <.001$	.110	.109	.726	.757	.780	17059.09	16816.33	
	B	$\chi^2(229) = 874.30, p <.001$	.109	.109	.731	.763	.785	17045.53	16802.77	
	C	$\chi^2(229) = 1017.90, p <.001$	.121	.114	.676	.698	.727	17191.80	16949.04	
	D	$\chi^2(229) = 1026.04, p <.001$	.121	.116	.680	.702	.730	17126.25	16883.49	
Single parents	Model SC	$\chi^2(251) = 857.32, p <.001$	.116	.101	.746	.784	.804	13867.24	13634.56	$\chi^2(2, 251) = 117.97, p <.001$
	EFA: Three factor	$\chi^2(249) = 685.67, p <.001$	.099	.074	.797	.844	.859	13705.97	13466.91	
	A	$\chi^2(229) = 696.06, p <.001$	.107	.098	.777	.820	.837	13380.99	13157.88	
	B	$\chi^2(229) = 679.99, p <.001$	.105	.095	.782	.826	.843	13411.42	13188.30	
	C	$\chi^2(229) = 804.53, p <.001$	.118	.100	.733	.769	.791	13476.39	13253.20	
	D	$\chi^2(229) = 788.22, p <.001$	.117	.102	.732	.770	.792	13507.68	13284.56	
Overweight people	Model SC	$\chi^2(251) = 903.90, p <.001$	.107	.081	.781	.814	.831	17744.21	17493.87	$\chi^2(5, 251) = 167.58, p <.001$
	EFA: Four factor	$\chi^2(246) = 654.44, p <.001$	.085	.050	.842	.881	.894	17521.89	17254.40	
	A	$\chi^2(229) = 730.13, p <.001$	.098	.083	.808	.844	.859	16959.32	16719.27	
	B	$\chi^2(229) = 737.18, p <.001$	.099	.083	.808	.843	.858	16982.80	16742.74	
	C	$\chi^2(229) = 738.80, p <.001$	.099	.077	.795	.832	.848	17154.79	16914.74	
	D	$\chi^2(229) = 756.64, p <.001$	.101	.079	.791	.827	.843	17180.15	16940.09	

Note. Model SC (i.e., Stigma Consciousness) is the preregistered two-factor model. We ran explorative factor analyses to investigate the underlying factor structure for each group, which revealed group specific factor solutions (indicated with EFA). Model A excludes item 9 of the anticipation subscale, model B excluded item 10 of the anticipation subscale, model C excludes item 7 of the anticipation subscale, model D excludes item 6 of the anticipation subscale.

Post-hoc analyses of the factor structure of the original Stigma Consciousness Questionnaire (Pinel, 1999)

Since the data-driven adjustments did not result in a significantly better fitting model, we focused instead on possible theory-driven adjustments. Specifically, we investigated whether the Stigma Consciousness Questionnaire (SCQ) developed by Pinel (1999) might independently be a more valid measure than our measure of stigma consciousness which included items of three measurement tools, the SCQ, the Stigmatization Scale and the Anticipated Stigma Scale. We believe this approach to be sensible, given that the SCQ has been most widely used in previous research on stigma consciousness and validated for a variety of groups (Pinel, 1999). We thus ran CFA with the items of the

original SCQ only, assuming two latent factors, stigma awareness and stigma anticipation, which were allowed to correlate. Each item was constrained to load on its respective factor. Since assumptions regarding normality were violated on sample and subsample level, we chose maximum likelihood with robust standard errors (MLR) as a robust estimation method for the CFA.

Model fit indices of the two-factor solution of the SCQ for the full sample as well as each subsample are reported in Table S.6, item loadings and covariances are reported in Table S.7. On sample level the model demonstrated marginal (RMSEA = .092; TLI = .876) to acceptable model fit (SRMR = .056; NFI = .056; CFI = .911). All items loaded significantly (p <.001) on their respective factor (Table S.6).

At subsample level, we observed substantial variability in model fit with Native Americans showing good fit values, while the homosexual and conservative subsamples displayed poor fit across all indices. Results suggest that the model's fit varies significantly, ranging from poor to good depending on the subsample and the specific fit indices considered, but interpretability of these differences is limited given the small subsample sizes.

Note, that because the adapted model includes only a subset of items used in the preregistered model, we cannot statistically compare the two models regarding their model fit. Thus, it remains unclear whether the improved fit indices of the two-factor model based on SCQ is due to a better representation of the data or simply a result of the reduced item number.

Table S.5
Reliability of SCQ variables

Group	Stigma Consciousness	Stigma Awareness	Stigma Anticipation
All groups	.76	.70	.74
African Americans	.80	.77	.72
Asian Americans	.78	.73	.71
Native Americans	.85	.84	.75
Latin Americans	.83	.82	.79
Alcoholics	.66	.62	.70
Poor people	.77	.72	.78

Unemployed people	.77	.63	.75
Overweight people	.82	.71	.83
Wheelchair users	.78	.69	.64
Transgender	.71	.69	.73
Homosexual people	.74	.61	.73
Conservatives	.67	.63	.67
Liberals	.66	.65	.66
Muslims	.70	.60	.61
Jews	.77	.70	.76
Atheists	.73	.67	.75
Old people	.75	.74	.75
Single Parents	.77	.59	.80

Table S.6

Model fit for SCQ two factor solution

**Group**	χ^2	*RMSEA*	*SRMR*	*NFI*	*TLI*	*CFI*
All	$\chi^2(26) = 901.81, p < .001$	.092	.056	.908	.876	.911
African American	$\chi^2(26) = 67.76, p < .001$	.083	.062	.908	.917	.940
Asian American	$\chi^2(26) = 69.27, p < .001$	.083	.063	.895	.904	.930
Native American	$\chi^2(26) = 32.82, p = .167$	.043	.042	.942	.982	.987
Latin American	$\chi^2(26) = 85.77, p < .001$	.099	.057	.906	.905	.932
Alcoholics	$\chi^2(26) = 74.65, p < .001$	.088	.065	.874	.879	.913
Poor people	$\chi^2(26) = 62.35, p < .001$	.076	.055	.901	.915	.939
Unemployed people	$\chi^2(26) = 41.69, p < .001$	.051	.050	.929	.960	.971
Wheelchair users	$\chi^2(26) = 56.17, p < .001$	.087	.057	.847	.874	.909

Transgender	$\chi^2(26) = 63.10, p <.001$	.085	.060	.859	.876	.910
Homosexual people	$\chi^2(26) = 111.44, p <.001$	.119	.080	.787	.757	.825
Conservatives	$\chi^2(26) = 105.05, p <.001$	.115	.097	.774	.745	.816
Liberals	$\chi^2(26) = 81.33, p <.001$	.095	.071	.829	.826	.874
Muslims	$\chi^2(26) = 92.68, p <.001$	.107	.072	.770	.748	.818
Jews	$\chi^2(26) = 77.04, p <.001$	.089	.052	.870	.873	.908
Atheists	$\chi^2(26) = 75.95, p <.001$	.089	.061	.875	.879	.913
Old people	$\chi^2(26) = 93.58, p <.001$	.105	.071	.847	.838	.883
Single parents	$\chi^2(26) = 57.69, p <.001$	.083	.051	.908	.925	.946
Overweight	$\chi^2(26) = 54.16, p <.001$	.069	.045	.931	.948	.962

Table S.7

Confirmatory factor analyses for two-factor solution of the stigma consciousness questionnaire (Pinel, 1999) per subsample

**Group**	**Covariance**	**Item**	**Factor 1 (Stigma Awareness)**		**Factor 2 (Stigma Anticipation)**	
			Item loading	R^2	***Item loading***	R^2
All groups	.492 (<.001)	1 (rec.)	.535 (<.001)	.286		
		2	.541 (<.001)	.293		
		3	.786 (<.001)	.618		
		4	.805 (<.001)	.648		
		5 (rec.)	.241 (<.001)	.058		
		14 (rec.)			.698 (<.001)	.488
		15 (rec.)			.779 (<.001)	.606
		16			.378 (<.001)	.143
		17 (rec.)			.761 (<.001)	.579
African American	.557 (<.001)	1 (rec.)	0.533 (<.001)	0.285		
		2	0.634 (<.001)	0.402		
		3	0.817 (<.001)	0.667		
		4	0.808 (<.001)	0.654		

		5 (rec.)	0.479 (<.001)	0.229		
		14 (rec.)			0.728 (<.001)	0.530
		15 (rec.)			0.888 (<.001)	0.788
		16			0.190 (.021)	0.036
		17 (rec.)			0.741 (<.001)	0.549
Asian American	.581 (<.001)	1 (rec.)	0.674 (<.001)	0.454		
		2	0.511 (<.001)	0.261		
		3	0.786 (<.001)	0.618		
		4	0.760 (<.001)	0.577		
		5 (rec.)	0.299 (<.001)	0.089		
		14 (rec.)			0.782 (<.001)	0.612
		15 (rec.)			0.777 (<.001)	0.604
		16			0.217 (.016)	0.047
		17 (rec.)			0.734 (<.001)	0.539
Native American	.725 (<.001)	1 (rec.)	0.808 (<.001)	0.652		
		2	0.735 (<.001)	0.540		
		3	0.787 (<.001)	0.619		
		4	0.874 (<.001)	0.764		
		5 (rec.)	0.439 (<.001)	0.192		
		14 (rec.)			0.563 (<.001)	0.317
		15 (rec.)			0.759 (<.001)	0.576
		16			0.448 (<.001)	0.201
		17 (rec.)			0.879 (<.001)	0.773
Latin American	.557 (<.001)	1 (rec.)	0.778 (<.001)	0.605		
		2	0.499 (<.001)	0.249		
		3	0.842 (<.001)	0.709		
		4	0.832 (<.001)	0.691		
		5 (rec.)	0.519 (<.001)	0.269		
		14 (rec.)			0.769 (<.001)	0.591
		15 (rec.)			0.865 (<.001)	0.747
		16			0.419 (<.001)	0.175
		17 (rec.)			0.748 (<.001)	0.560
Alcoholics	.219 (.035)	1 (rec.)	0.398 (.001)	0.158		
		2	0.577 (.001)	0.333		
		3	0.800 (<.001)	0.641		

		4	0.855 (<.001)	0.732		
		5 (rec.)	0.045 (.582)	0.002		
		14 (rec.)			0.735 (<.001)	0.540
		15 (rec.)			0.823 (<.001)	0.677
		16			0.199 (.033)	0.039
		17 (rec.)			0.741 (<.001)	0.550
Poor people	.459 (<.001)	1 (rec.)	0.607 (<.001)	0.368		
		2	0.515 (<.001)	0.265		
		3	0.841 (<.001)	0.707		
		4	0.718 (<.001)	0.515		
		5 (rec.)	0.328 (<.001)	0.108		
		14 (rec.)			0.668 (<.001)	0.446
		15 (rec.)			0.712 (<.001)	0.507
		16			0.533 (<.001)	0.284
		17 (rec.)			0.823 (<.001)	0.677
Unemployed people	.539 (<.001)	1 (rec.)	0.456 (<.001)	0.208		
		2	0.605 (<.001)	0.366		
		3	0.816 (<.001)	0.665		
		4	0.822 (<.001)	0.676		
		5 (rec.)	0.034 (.682)	0.001		
		14 (rec.)			0.740 (<.001)	0.547
		15 (rec.)			0.767 (<.001)	0.588
		16			0.402 (<.001)	0.162
		17 (rec.)			0.742 (<.001)	0.551
Wheelchair users	.788 (<.001)	1 (rec.)	0.658 (<.001)	0.433		
		2	0.378 (.004)	0.143		
		3	0.706 (<.001)	0.498		
		4	0.725 (<.001)	0.525		
		5 (rec.)	0.411 (<.001)	0.169		
		14 (rec.)			0.615 (<.001)	0.378
		15 (rec.)			0.795 (<.001)	0.631
		16			0.196 (.059)	0.038
		17 (rec.)			0.671 (<.001)	0.450
Transgender	.379 (.002)	1 (rec.)	0.752 (<.001)	0.565		
		2	0.355 (<.001)	0.126		

		3	0.586 (<.001)	0.344		
		4	0.764 (<.001)	0.584		
		5 (rec.)	0.425 (<.001)	0.181		
		14 (rec.)			0.444 (<.001)	0.197
		15 (rec.)			0.846 (<.001)	0.716
		16			0.467 (<.001)	0.219
		17 (rec.)			0.777 (<.001)	0.604
Homosexual people	.423 (.010)	1 (rec.)	0.515 (<.001)	0.266		
		2	0.348 (.022)	0.121		
		3	0.680 (<.001)	0.462		
		4	0.748 (<.001)	0.560		
		5 (rec.)	0.259 (.002)	0.067		
		14 (rec.)			0.724 (<.001)	0.524
		15 (rec.)			0.856 (<.001)	0.733
		16			0.348 (<.001)	0.121
		17 (rec.)			0.662 (<.001)	0.438
Conservatives	.211 (.187)	1 (rec.)	0.432 (<.001)	0.187		
		2	0.440 (<.001)	0.194		
		3	0.720 (<.001)	0.519		
		4	0.872 (<.001)	0.761		
		5 (rec.)	0.189 (.024)	0.036		
		14 (rec.)			0.565 (<.001)	0.319
		15 (rec.)			0.740 (.001)	0.548
		16			0.354 (.022)	0.126
		17 (rec.)			0.710 (<.001)	0.504
Liberals	.436 (<.001)	1 (rec.)	0.578 (<.001)	0.334		
		2	0.328 (<.001)	0.108		
		3	0.706 (<.001)	0.498		
		4	0.846 (<.001)	0.715		
		5 (rec.)	0.325 (<.001)	0.106		
		14 (rec.)			0.549 (<.001)	0.301
		15 (rec.)			0.653 (<.001)	0.427
		16			0.387 (.001)	0.150
		17 (rec.)			0.758 (<.001)	0.574
Muslims	.637 (.322)	1 (rec.)	0.569 (<.001)	0.324		

		2	0.279 (.413)	0.078		
		3	0.580 (.381)	0.336		
		4	0.558 (.369)	0.311		
		5 (rec.)	0.479 (<.001)	0.230		
		14 (rec.)			0.664 (<.001)	0.441
		15 (rec.)			0.739 (<.001)	0.547
		16			0.131 (.175)	0.017
		17 (rec.)			0.692 (<.001)	0.479
Jews	.522 (<.001)	1 (rec.)	0.653 (<.001)	0.427		
		2	0.449 (<.001)	0.202		
		3	0.745 (<.001)	0.555		
		4	0.743 (<.001)	0.552		
		5 (rec.)	0.265 (.001)	0.070		
		14 (rec.)			0.702 (<.001)	0.493
		15 (rec.)			0.780 (<.001)	0.608
		16			0.491 (<.001)	0.241
		17 (rec.)			0.669 (<.001)	0.447
Atheists	.380 (<.001)	1 (rec.)	0.656 (<.001)	0.430		
		2	0.454 (<.001)	0.206		
		3	0.749 (<.001)	0.562		
		4	0.864 (<.001)	0.747		
		5 (rec.)	0.044 (.594)	0.002		
		14 (rec.)			0.556 (<.001)	0.310
		15 (rec.)			0.699 (<.001)	0.489
		16			0.619 (<.001)	0.383
		17 (rec.)			0.768 (<.001)	0.590
Old people	.349 (.004)	1 (rec.)	0.565 (<.001)	0.319		
		2	0.548 (<.001)	0.300		
		3	0.795 (<.001)	0.631		
		4	0.756 (<.001)	0.571		
		5 (rec.)	0.406 (<.001)	0.165		
		14 (rec.)			0.674 (<.001)	0.455
		15 (rec.)			0.790 (<.001)	0.623
		16			0.436 (<.001)	0.190
		17 (rec.)			0.733 (<.001)	0.538

Single parents	.568 (<.001)	1 (rec.)	0.643 (<.001)	0.414		
		2	0.596 (<.001)	0.355		
		3	0.803 (<.001)	0.644		
		4	0.852 (<.001)	0.726		
		5 (rec.)	-0.280 (.014)	0.078		
		14 (rec.)			0.792 (<.001)	0.627
		15 (rec.)			0.792 (<.001)	0.627
		16			0.451 (<.001)	0.204
		17 (rec.)			0.825 (<.001)	0.680
Overweight people	.627 (<.001)	1 (rec.)	0.523 (<.001)	0.274		
		2	0.699 (<.001)	0.488		
		3	0.776 (<.001)	0.602		
		4	0.859 (<.001)	0.738		
		5 (rec.)	0.161 (.037)	0.026		
		14 (rec.)			0.759 (<.001)	0.576
		15 (rec.)			0.783 (<.001)	0.614
		16			0.622 (<.001)	0.386
		17 (rec.)			0.803 (<.001)	0.645

Note. A list of all items with item number and wording is reported in Appendix S1, Table S.5.

Conclusion regarding the Stigma Consciousness measure

Based on extensive literature research, we had employed a relatively large number of items from three established measures to form a stigma consciousness scale that can be differentiated in two subscales: stigma awareness and stigma anticipation. However, confirmatory factor analyses revealed that the preregistered two-factorial model did not fit the data well and data-driven post hoc modifications did not reveal better fitting factor solutions which could be equally applied to each subsample. The results did also not support the anticipated distinction of stigma consciousness into stigma awareness and stigma anticipation, suggesting that either the items did not adequately capture the separate latent variables, or that the two constructs may not be empirically inseparable, as assumed by Pinel (1999).

Based on these results we conducted post-hoc non-preregistered confirmatory factor analyses of the original SCQ scale developed by Pinel (1999), testing the hypothesized two-factor solution (stigma awareness and stigma anticipation). Results revealed good to acceptable fit indices for the sample as well as within each subsample.

With regard to our research goal to examine the relationship of stigma consciousness and individual- and group-level predictors, we included only the SCQ items for all further analyses. Given the only acceptable model fit and the variation in model fit between subsamples, we further decided to not differentiate stigma consciousness into two subcomponents. This approach allows us to use a measurement tool validated in prior research and enables us to compare our results to prior research which used this measure in various stigmatized groups (Pinel, 1999; Pinel, 2004).

References

Bernaards, C. A., & Jennrich, R. I. (2005). Gradiant Projection Algorithms and Software for Arbitrary Rotation Criteria in Factor Analysis. Educational and Psychological Measurement, 65(5), 676-696. https://doi.org/10.1177/0013164404272507

Harvey, R. D. (2001). Individual differences in the phenomenological impact of social stigma. *The Journal of Social Psychology, 141*(2), 174-189. https://doi.org/10.1080/00224540109600545

Fox, J., & Weisberg, S. (2019). *An R Companion to applied regression*, (3rd edition). Sage publications. https://www.john-fox.ca/Companion/

Fox, J., Weisberg, S., & Price, B. (2022). CarData: Companion to applied regression data sets. *CRAN: Contributed packages*, https://r-forge.r-project.org/projects/car

Korkmaz, S., Gokusluk, D. & Zarasiz, G. (2014). MVN: An R package for assessing multivariate normality. *The R Journal, 6*(2), 151-162.

Papadakis, M., Tsagris, M., & Fafalios, S. (2023). Rfast: A collection of efficient and extremely fast R functions. *CRAN: Contributed packages*, https://github.com/rfastofficial/rfast

Pinel, E. C. (1999). Stigma consciousness: The psychological legacy of social stereotypes. *Journal of Personality and Social Psychology, 76*(1), 114-125. https://doi.apa.org/doi/10.1037/0022-3514.76.1.114

Pinel, E. C. (2004). You're just saying that because I'm a woman: Stigma consciousness and attributions to discrimination. *Self and Identity, 3*(1), 39-51. https://doi.org/10.1080/13576500342000031

Quinn, D. M., & Chaudoir, S. R. (2009). Living with a concealable stigmatized identity: The impact of anticipated stigma, centrality, salience, and cultural stigma on psychological distress and health. *Journal of Personality and Social Psychology, 97*(4), 634-651. https://doi.apa.org/doi/10.1037/a0015815

Rosseel, Y. (2012). Lavaan: An R package for structural equation modeling. Journal of Statistical Software, 48(2), 1-36. https://doi.org/10.18637/jss.v048.i02

Wickham, H., Averick, M., Bryan, J., Chang, W., McGowan, L. D., François, R., Grolemund, G., Hayes, A., Henry, L., Hester, J., Kuhn, M., Pedersen, T. L., Miller, E., Bache, S. M., Müller, K., Ooms, J., Robinson, D., Seidel, D. P., Spinu, V., Takahashi, K., Vaughan, D., Wilke, C., Woo, K., & Yutani, H. (2019). Welcome to tidyverse. *Journal of Open Source Software, 4*(43), 1686. https://doi.org/10.21105/joss.01686

Wickham, H., François, R., Henry, L., Müller, K., & Vaughan, D. (2023a). Dplyr: A grammar of data manipulation. *CRAN: contributed packages*, https://doi.org/10.32614/CRAN.package.dplyr

Wickham, H., Miller, E., & Smith, D. (2023b). Haven: Import and export 'SPSS', 'Stata' and 'SAS' Files. *CRAN: contributed packages*, https://doi.org/10.32614/CRAN.package.haven

William, R. (2024). Psych: Procedures for psychological, psychometric, and personality research. *CRAN: contributed packages*, https://doi.org/10.32614/CRAN.package.psych
